# eIF2β zinc-binding domain interacts with the eIF2γ subunit through the guanine nucleotide binding interface to promote Met-tRNA_i_^Met^ binding

**DOI:** 10.1042/BSR20240438

**Published:** 2024-07-05

**Authors:** Aranyadip Gayen, Pankaj V. Alone

**Affiliations:** 1 School of Biological Sciences, National Institute of Science Education and Research Bhubaneswar, P.O Jatni, Khurda 752050, India; 2 Homi Bhabha National Institute (HBNI), Anushakti Nagar, Mumbai 400094, India

**Keywords:** eIF2, Met-tRNAiMet, Ribosome, ternary complex, Translation initiation

## Abstract

The heterotrimeric eIF2 complex consists of a core eIF2γ subunit to which binds eIF2α and eIF2β subunits and plays an important role in delivering the Met-tRNA_i_^Met^ to the 40S ribosome and start codon selection. The intricacies of eIF2β-γ interaction in promoting Met-tRNA_i_^Met^ binding are not clearly understood. Previously, the zinc-binding domain (ZBD) eIF2β^S264Y^ mutation was reported to cause Met-tRNA_i_^Met^ binding defect due to the intrinsic GTPase activity. We showed that the eIF2β^S264Y^ mutation has eIF2β-γ interaction defect. Consistently, the eIF2β^T238A^ intragenic suppressor mutation restored the eIF2β-γ and Met-tRNA_i_^Met^ binding. The eIF2β-ZBD residues Asn252Asp and Arg253Ala mutation caused Met-tRNA_i_^Met^ binding defect that was partially rescued by the eIF2β^T238A^ mutation, suggesting the eIF2β-ZBD modulates Met-tRNA_i_^Met^ binding. The suppressor mutation rescued the translation initiation fidelity defect of the eIF2γ^N135D^ SW-I mutation and eIF2β^F217A/Q221A^ double mutation in the HTH domain. The eIF2β^T238A^ suppressor mutation could not rescue the eIF2β binding defect of the eIF2γ^V281K^ mutation; however, combining the eIF2β^S264Y^ mutation with the eIF2γ^V281K^ mutation was lethal. In addition to the previously known interaction of eIF2β with the eIF2γ subunit via its α1-helix, the eIF2β-ZBD also interacts with the eIF2γ subunit via guanine nucleotide-binding interface; thus, the eIF2β-γ interacts via two distinct binding sites.

## Introduction

**Figure 1:**
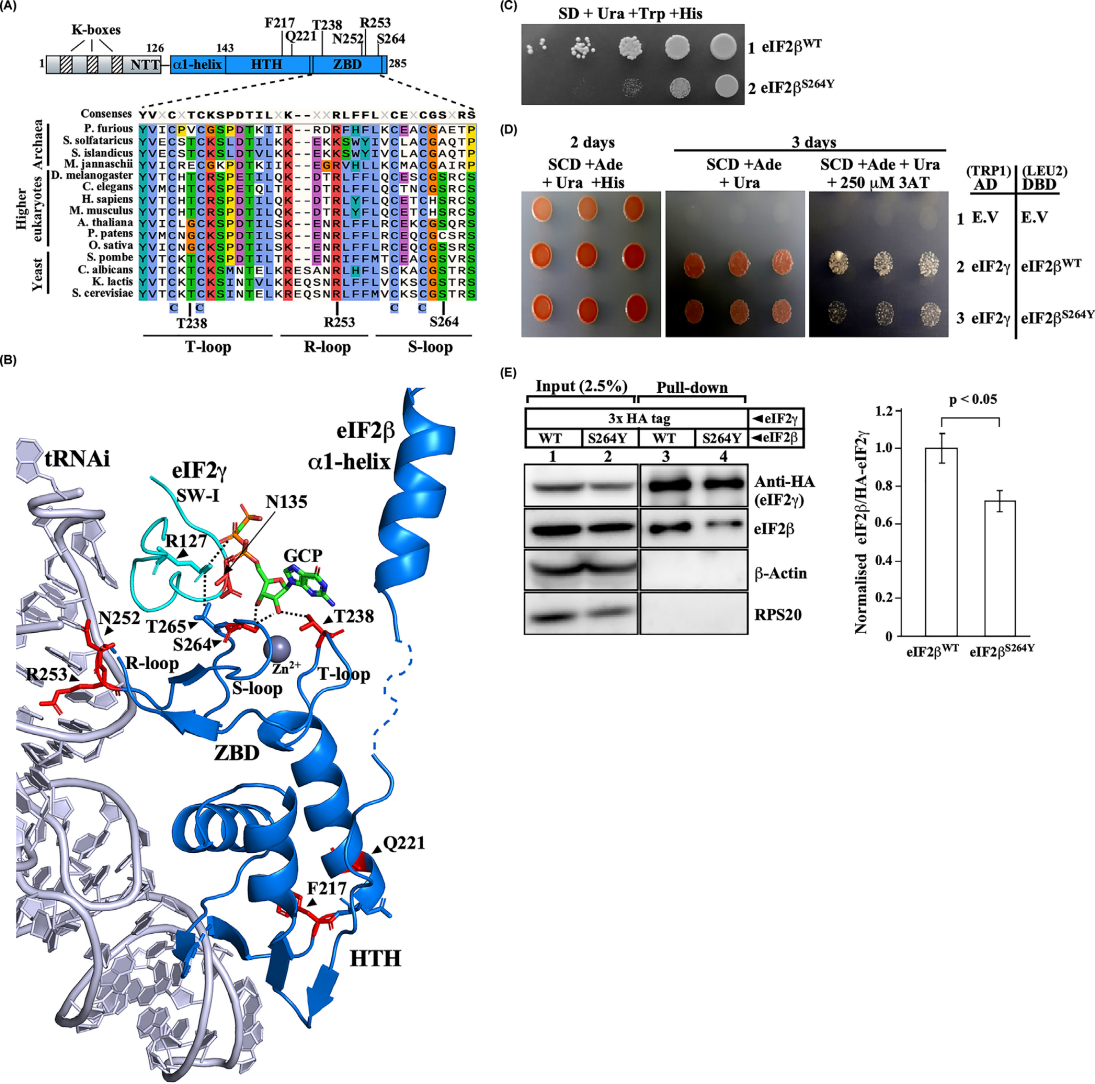
eIF2β^S264Y^ zinc-binding domain mutation causes eIF2γ binding defect (**A,B**) Schematic representation, sequence homology, and structure of eIF2β. (A) The eIF2β is divided into an N-terminal tail (NTT) containing three K-boxes, the middle region consisting of α1-helix, the C-terminal region consisting of helix-turn-helix, and a zinc-binding domain. Different mutations in the eIF2β subunit are indicated. Multiple sequence alignment of eIF2β-ZBD from the indicated organisms belonging to archaea, yeast, and higher eukaryotes was done using the Clustal-omega algorithm. The position of the four conserved cystines, Thr238 and Ser264 residues, are shown at the bottom. The T-loop, R-loop, and S-loop indicate different regions of the eIF2β-ZBD. (B) The cryoEM structure of partial yeast 48S PIC (6FYX) [5] viewed using PYMOL software [10] showing eIF2β (blue), Met-tRNAi (grey), eIF2γ switch-I region (SW-I; cyan), Zn^2+^ ion (sphere; grey) and a GCP molecule. The eIF2β-ZBD’s T-loop, R-loop, and S-loop are indicated. Residues that were mutated in this study are shown in red color sticks. Polar interactions of Ser264 and Thr238 residues with the guanine nucleotide are shown with a dotted line. The polar interaction of eIF2γ Arg127 residue with the eIF2β Thr265 and α-PO_4_ of guanine nucleotide is indicated by a dotted line. (**C**) Growth analysis. Yeast strain YP896 (*his4Δ, sui3Δ*) carrying single copy eIF2β^WT^ (A1451) or eIF2β^S264Y^ (A1446) plasmids were grown overnight, serially diluted, and spotted on SD media plate supplemented with uracil, tryptophane and histidine and incubated at 30°C for 2–3 days. (**D**) Analysis of eIF2β-eIF2γ interaction by yeast two-hybrid. The eIF2γ^WT^ protein was fused with the Activating domain (AD) (A1242), and the eIF2β^WT^ (A1237) or eIF2β^S264Y^ (A1238) mutant proteins were fused with the DNA binding domain (DBD) and transformed into the yeast strain (YP930) along with the empty vector (EV) (A643) and (A1236), serially diluted and spotted on SCD adenine plate supplemented with uracil or SCD supplemented with uracil and 250 μM 3-amino triazole (3-AT). (**E**) Co-Immunoprecipitation assay. Yeast strain YP912 (*gcd11Δ, sui3Δ*) carrying *GCD11* gene encoding N-terminal 3xHA eIF2γ transformed with either a single copy eIF2β^WT^ (A1451) or eIF2β^S264Y^ (A1446) plasmids were grown overnight and harvested at OD_600_ ∼ 0.8. The WCE (300 μg) was incubated with anti-HA antibody agarose beads, and the eIF2γ protein was co-immunoprecipitated. One-fifth of the beads were analysed by Western blot using anti-HA, anti-RPS20, anti-β-actin and anti-eIF2β antibodies. The input lanes contain 2.5% of the WCE. The densitometric quantitation of the blot is shown on the right. Statistical differences were determined by one-way ANOVA analysis. The error bar shows the standard deviation obtained from at least three biological replicates.

In the three domains of life, translation initiation is a critical phase in which the AUG start codon is selected to establish an open reading frame (ORF) for protein biosynthesis. The archaea and eukaryotes use heterotrimeric a/eIF2 consisting of the GTPase core a/eIF2γ subunit to which binds a/eIF2α and a/eIF2β subunit, and along with the GTP and Met-tRNA_i_^Met^ forms a ternary complex (TC), which delivers the Met-tRNA_i_^Met^ to the P-site of the 40S ribosome [1,2]. The eIF2γ subunit is made of G-domain (residues 1-309), domain II (residues 310-412), and domain III (residues 413-529). The eIF2γ G-domain has characteristic motifs for the guanine nucleotide binding and switch-I (SW-I) and switch-II (SW-II) regions for the GTP hydrolysis. The G-domain and domain II are packed together to form a Met-tRNA_i_^Met^ binding pocket [3,4]. The eIF2α subunit is made of an N-terminal domain (residues 1-90), middle domain (residues 91-174), and the C-terminal domain (residues 182-265) and shows multiple contacts with the Met-tRNA_i_^Met^ and plays an important regulatory role in translation initiation control (integrated stress response in the higher eukaryotes) via phosphorylation of its Ser51 residue [5–7]. The eIF2β subunit has a long unstructured N-terminal tail (residues 1-126), followed by α1-helix (residues 128-143), a helix turn helix (HTH) domain (residues 155-234), and at the C-terminal end, a zinc-binding domain (ZBD) (resides 235-270) which is made of three distinct loops [5]. For simplicity, we are referring to the eIF2β-ZBD loop region from amino acids 235-244 containing residue T238 as a T-loop, the region from 245-256 containing residue R253 as a R-loop, and the loop region from 257-270 containing residue S264 as an S-loop (Figure 1A). The eIF2β subunit is reported to show guanine nucleotide dissociation inhibitor (GDI) activity and also interacts with the GTPase-activating protein (GAP) eIF5 subunit by the N-terminal lysine (K) -boxes [8,9].

In the eukaryotic translation initiation process, translation initiation factor eIF1 binds near the P-site, eIF1A binds at the A-site, and the eIF3 is assembled on the solvent side of the 40S ribosome. The TC and eIF5 are recruited to this complex to form a 43S pre-initiation complex (PIC). The 43S PIC then binds to the activated mRNA-eIF4F complex to form a 48S complex and scans the mRNA from the 5′ to 3′ direction in search of the AUG start codon [11]. During the scanning process, the eIF5 Arg15 residue is proposed to interact with the eIF2γ G-domain to hydrolyse the GTP molecule to GDP + P_i_; however, the P_i_ release is blocked by the eIF1 [12–14]. When the AUG start codon enters the 40S ribosomal P-site, the codon:anti-codon interaction causes significant structural rearrangement in the 48S scanning complex. The eIF1 is released from the 40S ribosomal P-site and an attendant P_i_ release from the eIF2γ subunit, the 40S ribosomal head rotates from the ‘Open’ conformation to ‘Closed’ state, and the Met-tRNA_i_^Met^ conformation changes from the P_OUT_ to P_IN_ state [15]. Mutations in the translation initiation factors that disrupt these processes and prematurely insert the Met-tRNA_i_^Met^ in the P_IN_ state even in the absence of the AUG start codon at the P-site often relax the stringency of start codon recognition (suppressor of initiation codon; Sui¯ phenotype). For example, the eIF2γ^N135D/A382V^ and eIF2β^S264Y^ mutations show defects in Met-tRNA_i_^Met^ binding and enhance translation initiation from the UUG codon of the *His4^UUG^* allele [15–18]. It is important to emphasize that the Sui¯ phenotype is caused by the delivery of Met-tRNA_i_^Met^ in an altered conformation (P_IN_) and the attendant premature dissociation of the eIF1 from the scanning 48S ribosomal complex rather than the premature dissociation of Met-tRNA_i_^Met^ [15,17,18]. The defect in the fidelity of start codon selection can have a pleiotropic effect on the cellular proteome and adversely affect cellular physiology [19].

The a/eIF2γ subunit is structurally and functionally homologous to the bacterial elongation factor EF-Tu and the a/eEF-1A, which binds to the charged elongator tRNAs and delivers them to the A-site of the 70S/80S ribosome in the elongation phase of protein synthesis without the additional support of any other proteins [20]. Interestingly, the eIF2αβγ orthologs are absent in the eubacteria, where a single polypeptide initiation factor IF2 is responsible for binding Met-tRNA_i_^Met^ to the 30S ribosome [21]. In the absence of the eIF2γ ortholog in eubacteria, it could be possible that in archaea or eukaryotes, the a/eIF2γ might have evolved from the EF-Tu/aEF-1A. Whereas in the EF-Tu/aEF-1A, the G-domain, domain II, and domain III are packed together to form an elongator tRNA (tRNA_e_) binding pocket, in the case of eIF2γ subunit, the Met-tRNA_i_^Met^ is rotated 165° to interact with the G-domain and domain II interface [3,22]. In this orientation, the Met-tRNA_i_^Met^ contacts with the eIF2γ subunit are far less (∼32%) compared with the elongator tRNA_e_ (100%) bound to the EF-Tu/EF-1A [3,23]. The eIF2α and eIF2β subunits contribute the rest of the contacts with the Met-tRNA_i_^Met^. The eIF2α and eIF2β subunit may have evolved to associate with the eIF2γ subunit and provide additional contact sites by sandwiching the Met-tRNA_i_^Met^ between them and stabilizing the Met-tRNA_i_^Met^ in the TC. In the present study, we have explored the intricacies of eIF2β-γ interaction, especially the role of eIF2β-ZBD in binding with the core eIF2γ subunit and how it promotes the Met-tRNA_i_^Met^ binding. Our study revealed that the Ser264Tyr substitution in the ZBD S-loop led to the eIF2β binding defect. Next, we screened for the intragenic suppressor mutation that could rescue the growth defect of the eIF2β^S264Y^ mutation. Interestingly, the eIF2β^T238A^ suppressor mutation isolated in the T-loop rescues the eIF2β-γ binding defect; it partially rescues the Met-tRNA_i_^Met^ binding defect and the Sui¯ phenotype of the eIF2β^S264Y^ mutant. In addition to the previously known interaction of eIF2β with the eIF2γ subunit via its α1-helix, we showed that the eIF2β-ZBD also interacts with the eIF2γ subunit via guanine nucleotide-binding interface and promotes Met-tRNA_i_^Met^ binding to the TC.

## Results

### The eIF2β^S264Y^ zinc-binding domain mutation causes eIF2γ binding defect

The eIF2β^S264Y^ mutation was extensively used to study the eIF2 function [3,5,18,24–33]. However, mechanistic insight into the working of eIF2β^S264Y^ mutation is not clearly understood. The eIF2β^S264Y^ mutation was proposed to cause eIF5-independent intrinsic GTPase activity in the ternary complex to release the Met-tRNA_i_^Met^ prematurely [24]. It could be possible that the eIF2β^S264Y^ mutation may have other defects that interfere with the Met-tRNA_i_^Met^ binding. Structural data of the TC suggest that the eIF2β subunit binds to the eIF2γ subunit via α1-helix. The eIF2β-ZBD residues Thr238 and Ser264 also show polar interaction with the ribose sugar moiety (2′OH and 3′OH) of GDP/GTP bound to the eIF2γ subunit (Figure 1B) [5]. However, the importance of this interaction was never tested. The eIF2β^S264^ residue is relatively conserved in the yeast and higher eukaryotes (Figure 1A). We predicted that the eIF2β^S264Y^ mutation may alter the ZBD conformation and affect eIF2β-γ interaction. Consistently, the eIF2β^S264Y^ mutation showed a severe slow-growth (Slg¯) phenotype when the eIF2β^WT^ was evicted (Figure 1C). We used the yeast two-hybrid assay to test whether the eIF2β^S264Y^ mutation shows an *in vivo* binding defect with the eIF2γ subunit (see Materials and Methods). The eIF2γ^WT^ and eIF2β^S264Y^ mutant pair showed a slow growth phenotype on the SCD supplemented with uracil and 250 μM 3-AT plate (Figure 1D, row 3), indicating eIF2γ^WT^-eIF2β^S264Y^ interaction defect. To confirm that the eIF2β^S264Y^ mutation has an interaction defect with the eIF2γ protein, we conducted an *in vivo* co-immunoprecipitation experiment. We added a 3xHA-tag to the N-terminal end of the eIF2γ protein, and using anti-HA antibody agarose beads the eIF2γ protein was pulled down from the whole-cell extract (WCE) of the wild-type and eIF2β^S264Y^ mutant yeast strains and analysed by the Western blot using anti-HA and anti-eIF2β antibodies. Consistent with the yeast two-hybrid data, the eIF2β^S264Y^ mutant showed a 30% binding defect with the eIF2γ subunit (Figure 1E). These data suggest that in addition to the eIF2β subunit binding to the eIF2γ-G domain via its α1-helix, the eIF2β-ZBD is also critically contributing to the interaction via the guanine nucleotide-binding interface.

### Isolation and characterization of eIF2β^S264Y^ intragenic suppressor mutation

**Figure 2:**
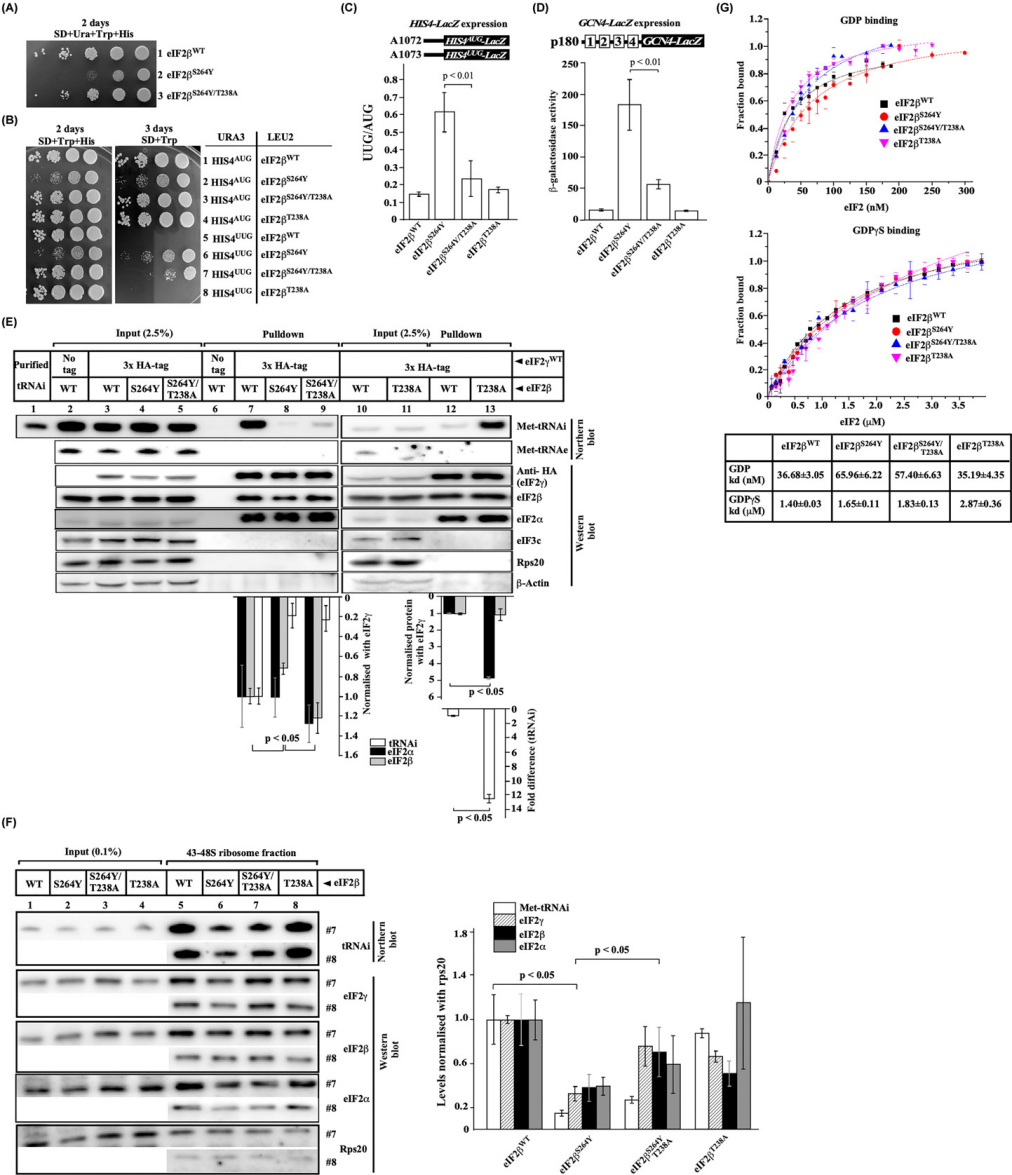
Phenotypic and biochemical analysis of eIF2β^S264Y^ and its intragenic suppressor mutation eIF2β^S264Y/T238A^ (**A**) Growth analysis. Yeast strain YP896 (*his4Δ, sui3Δ*) carrying plasmid-borne eIF2β^WT^ (A1451), eIF2β^S264Y^ (A1446), or eIF2β^T238A/S264Y^ (A1259) double mutant gene were grown overnight, serially diluted, and spotted on SD plate supplemented with uracil, tryptophane and histidine and incubated at 30°C for 2 days. (**B**) Analysis of Sui¯ phenotype. Yeast strain YP896 (*his4Δ, sui3Δ*) carrying plasmid-borne eIF2β^WT^ (A1451), eIF2β^S264Y^ (A1446), eIF2β^T238A^ (A1260) or eIF2β^T238A/S264Y^ (A1259) double mutant gene were transformed with either *HIS4^AUG^* (A839) or, *HIS4^UUG^* (A840) constructs and grown overnight, serially diluted, and spotted on SD plate supplemented with tryptophane and histidine or SD plate supplemented with tryptophane and incubated for 2–3 days at 30°C. (**C**) Analysis of *HIS4-lacZ* expression. Yeast strain YP896 (*his4Δ, sui3Δ*) carrying plasmid borne eIF2β^WT^ (A1451), eIF2β^S264Y^ (A1446), eIF2β^T238A^ (A1260) or eIF2β^T238A/S264Y^ (A1259) double mutant gene were transformed with either A1072 (GAPDH_prom__His4^AUG^_lacZ) or, A1073 (GAPDH_prom__His4^UUG^_lacZ) plasmid constructs and grown on the SCD media supplemented with tryptophane and histidine and harvested at OD_600_ ∼ 0.8. The whole cell extract was prepared and β-galactosidase activity (nmol of O-nitrophenyl-β-D-galactopyranoside cleaved per min per mg) was measured and the resultant values were plotted as UUG/AUG ratio as described previously [36]. (**D**) Analysis of *GCN4-lacZ* expression. Yeast strain YP896 (*his4Δ, sui3Δ*) carrying plasmid borne eIF2β^WT^ (A1451), eIF2β^S264Y^ (A1446), eIF2β^T238A^ (A1260) or eIF2β^T238A/S264Y^ (A1259) double mutant gene were transformed with *GCN4-lacZ* construct (p180). The measurement of β-galactosidase activity was done as per (**C**). (**E**) Co-Immunoprecipitation assay. Yeast strain YP912 (*gcd11Δ*, *sui3Δ*) carrying plasmid borne N-terminal 3xHA-tag eIF2γ subunit (A1404) and derivatives of eIF2β [eIF2β^WT^ (A1451), eIF2β^S264Y^ (A1446), eIF2β^T238A^ (A1260) or eIF2β^T238A/S264Y^ (A1259) double mutant] gene were subjected to Co-IP as described in Figure 1E. The Co-IP beads were analysed by Western blot using anti-HA, anti-eIF2β, anti-eIF2α, anti-eIF3c, anti-actin, and anti-rps20 antibodies and by Northern blot using a probe specific to initiator Met-tRNA_i_ or elongator Met-tRNA_e_. The input lanes contain 2.5% of the WCE. (**F**) Analysis of TC on the formaldehyde cross linked 48S ribosome. Yeast strain YP912 carrying derivatives of eIF2β mutant as per (**E**) were subjected to 1% HCHO cross-linking as described in Materials and Methods. Fractions #7 and #8 containing 40-48S ribosomes were analysed by Western and Northern blots as per (**E**). The input lanes contain 0.1% of A_260_ ∼ 20 Units. The graph (right) shows the densitometric quantitation of the TC on the 40S ribosomes from at least three biological replicates. (**G**) Analysis of guanine nucleotide binding. GDP-BODIPY and GDPγS-BODIPY were titrated with the derivative of purified eIF2 [eIF2β^WT^, eIF2β^S264Y^, eIF2β^T238A^ or eIF2β^T238A/S264Y^ double mutant]. The fluorescence anisotropy values were converted to fraction bound and plotted using Hill’s equation (Origin software). The *K*_d_ values for GDP and GDPγS for the WT or mutant proteins were determined from the three independent experiments and summarized in the table below (± standard deviations). Statistical differences were determined by one-way ANOVA analysis. The error bar shows the standard deviation obtained from the three biological replicates.

To gain further insights into the function of eIF2β-ZBD and its involvement in the TC complex formation, we screened for intragenic suppressors of the eIF2β^S264Y^ mutation (see Supplementary Data). We focused our work on the suppressor mutation that converts eIF2β-Thr238 to Ala in the ZBD (T-loop) (Figure 2A, row 3 and Figure 1B). The eIF2β^T238^ residue is relatively conserved in yeast and higher eukaryotes, except in plants, where Thr is replaced with Gly (Figure 1A). The eIF2β^S264Y^ mutation shows the Sui¯ phenotype [34,35]. To test if the eIF2β^S264Y/T238A^ suppressor mutation suppresses the Sui¯ phenotype of eIF2β^S264Y^ mutation, we used YP896 yeast strain (*his4Δ, sui3Δ*) containing a plasmid-borne copy of either eIF2β^WT^, eIF2β^S264Y^, eIF2β^S264Y/T238A^ or eIF2β^T238A^ genes and transformed with a plasmid-borne copy of either wild type *HIS4^AUG^* or *HIS4^UUG^* allele where the AUG codon is mutated to AUU, thus the third codon (UUG) is used as a start codon [11]. The eIF2β^WT^ did not initiate translation from the *HIS4^UUG^* construct and did not grow, whereas the eIF2β^S264Y^ mutant could initiate translation from the *HIS4^UUG^* construct and grew on the minus histidine plate (Sui¯ phenotype) (Figure 2B, rows 5 and 6). However, whereas the eIF2β^S264Y/T238A^ mutation partially suppressed the Sui¯ phenotype (Ssu¯ phenotype) of the eIF2β^S264Y^ mutant, the eIF2β^T238A^ mutation did not show Sui¯ phenotype (Figure 2B, row 7 and 8). Consistently, the *HIS4-LacZ* reporter assay showed higher initiation from the UUG codon by the eIF2β^S264Y^ mutant, whereas the eIF2β^S264Y/T238A^ double mutant partially suppressed the UUG codon utilization, and the eIF2β^T238A^ mutant seldom utilized the UUG as a start codon (Figure 2C).

The *GCN4* mRNA translation is very sensitive to the levels of TC availability for the translation initiation process, and it is regulated by the four upstream short open reading frames (uORFs 1-4) [11,37,38]. In normal conditions, the TC levels are high, and the 48S scanning ribosome complex translates uORF1; however, after the translation termination, the 40S ribosome stays bound to the *GCN4* mRNA to scan downstream and translate the inhibitory uORF3 or uORF4 and dissociate without reaching the main *GCN4* ORF. In stress or starvation conditions, the TC levels are low, and the scanning 40S ribosome skips the translation of downstream inhibitory uORF4 and translates the main *GCN4* ORF due to the late recruitment of the TC [11]. The *GCN4-LacZ* reporter assay was used to check the levels of TC in the eIF2β mutants. Consistency with its Met-tRNA_i_^Met^ and eIF2β binding defects, the eIF2β^S264Y^ mutant showed high levels of β-galactosidase activity (Gcd¯ phenotype), whereas the eIF2β^S264Y/T238A^ double mutant partially suppressed the Gcd¯ phenotype and the eIF2β^T238A^ mutation did not show Gcd¯ phenotype (Figure 2D). Collectively, these data suggest that the eIF2β^T238A^ intragenic suppressor mutation partially suppresses the Sui¯ and Gcd¯ phenotype of the eIF2β^S264Y^ and rescued the growth defect, possibly by restoring eIF2β binding to the eIF2γ subunit (see below).

To confirm whether the eIF2β mutants have Met-tRNA_i_^Met^ and eIF2γ binding defects, we performed an *in vivo* co-immunoprecipitation experiment. As described in the previous section, the 3xHA tagged eIF2γ was pulled down from the WCE of different eIF2β mutants, and it was analysed by the Northern blot using an oligonucleotide probe against Met-tRNA_i_ or Met-tRNA_e_ and Western blot using antibodies specific to HA-tag (eIF2γ), eIF2β, eIF2α, eIF3c, Rps20, and β-actin. Compared with the WT, the eIF2β^S264Y^ mutant showed ∼30% binding defect with the eIF2γ subunit and a severe (∼80%) binding defect with the Met-tRNA_i_^Met^ (Figure 2E, lanes 7 and 8). The absence of eIF3c and the 40S ribosomal protein Rps20 in the pulldown suggests that we analysed free TC unassociated with the multi-factorial complex or 40S ribosome. Notably, the elongator Met-tRNA_e_ does not interact with the eIF2 complex. Remarkably, whereas the eIF2β^T238A^ single mutant binds to the eIF2γ subunit near WT levels, the Met-tRNA_i_^Met^ and eIF2α subunit binding affinity was observed to be ∼12.5-fold and ∼4.5-fold higher, respectively (Figure 2E, lane 13). Co-IP of the 3xHA-tagged eIF2β subunit showed ∼3.5-fold and 1.3-fold higher binding affinity for the Met-tRNA_i_^Met^ and eIF2α subunit, respectively (Supplementary Figure S1). A previous report suggests that the Met-tRNA_i_^Met^ and eIF2α subunit binding sites are in close proximity on the eIF2γ-Domain II, and the eIF2α subunit contributes to the Met-tRNA_i_^Met^ binding to the eIF2γ subunit [4]. Our data reinforce the notion that the eIF2β^T238A^ substitution mutation alters the ZBD structure to strengthen the Met-tRNA_i_^Met^ binding affinity with the eIF2γ subunit and by its proximity to the eIF2α subunit also enhance the eIF2α-γ binding affinity.

In the eIF2β^S264Y/T238A^ double mutant, the binding defect of the mutant eIF2β with the eIF2γ subunit was rescued. However, the eIF2β^S264Y/T238A^ double mutant did not fully rescue the Met-tRNA_i_^Met^ binding defect (Figure 2E, lane 9). It is possible, in the eIF2β^S264Y/T238A^ double mutation, the Ser264 to Tyr substitution may continuously interfere with the Met-tRNA_i_^Met^ binding, even though the Thr238 to Ala substitution restores the eIF2β binding. Surprisingly, the eIF2β^S264Y/T238A^ double mutation partially suppressed the Gcd¯ phenotype, even though it showed a severe Met-tRNA_i_^Met^ binding defect with the TC. It is likely that in the eIF2β^S264Y/T238A^ double mutation, the Met-tRNA_i_^Met^ interaction with the eIF2 complex is weak as captured by this assay; however, the Met-tRNA_i_^Met^ may have a relatively stable interaction when associated with the 48S scanning ribosomal complex. To test this hypothesis, we treated the yeast cells with 1% formaldehyde to cross-link and stabilize the TC interaction on the 40S ribosome, and using a 15%-40% sucrose density gradient, we fractionated the translation initiation complex and performed Northern and Western blotting. Consistent with our hypothesis, in the eIF2β^S264Y^ mutant, the TC associated with the 40S ribosomal subunit was extremely low, whereas in the eIF2β^S264Y/T238A^ double mutation, the levels of the eIF2αβγ complex associated with the 40S ribosomal subunit significantly improved along with a modest improvement in the Met-tRNA_i_^Met^ bound to this complex (Figure 2F and Supplementary Figure S2). Interestingly, the eIF2β^T238A^ single mutant showed near WT levels of Met-tRNA_i_^Met^ bound to the 43-48S ribosomal complex, in contrast to the ∼12.5-fold high Met-tRNA_i_^Met^ interaction observed in the pull-down experiment (Figure 2E, lane 13 and Figure 2F, lane 8). These observations suggest that eIF2β^T238A^ mutation augments the eIF2α and Met-tRNA_i_^Met^ binding affinity with the core eIF2γ subunit, and makes the free TC more stable and capable of binding the 40S ribosome comparable to the WT levels.

The eIF2γ G-domain has characteristic motifs for the guanine nucleotide binding. Our mutation analysis suggests that the eIF2β-ZBD interacts with the eIF2γ subunit through the guanine nucleotide-binding interface. To check if the eIF2β-ZBD mutations affect the guanine nucleotide binding affinity of the eIF2 complex, we added 8xHis-tag to the eIF2γ subunit and purified WT or the mutant eIF2 complex from the yeast strain using a method described previously [17]. Interestingly, the level of the eIF2β^S264Y^ subunit was 20% lower in the purified mutant eIF2 complex (Supplementary figure S3), consistent with the eIF2β^S264Y^ binding defect observed in the Co-IP assay (Figures 1E and 2E). Next, we used fluorescent anisotropy to calculate the guanine nucleotide binding affinity using fluorescently labeled GDP or GDPγS with increasing concentrations of the purified eIF2 WT or the mutant proteins. Our result suggests the WT eIF2 binding affinity for GDP is 36.68 nM, consistent with the previously reported data [39]. The GDP binding affinity for the eIF2β^S264Y^ and eIF2β^S264Y/T238A^ double mutant was ∼1.7-fold and ∼1.5-fold lower than the WT, respectively, whereas the eIF2β^T238A^ mutation showed no change in GDP binding affinity (Figure 2G). The calculated WT eIF2 binding affinity for GDPγS was 1.40 μM, consistent with the previously reported data [39]. The eIF2β^S264Y^ and eIF2β^S264Y/T238A^ double mutant showed ∼1.1-fold and ∼1.17-fold lower GDPγS binding affinity than the WT protein. Although the eIF2β^T238A^ mutation showed a ∼2-fold decrease in GDPγS affinity, it showed no genetic or biochemical defects (Figure 2). These results suggest no significant changes in guanine nucleotide binding affinity difference with the eIF2 mutant proteins.

### The eIF2β-ZBD R-loop is critical for the Met-tRNA_i_^Met^ binding

**Figure 3:**
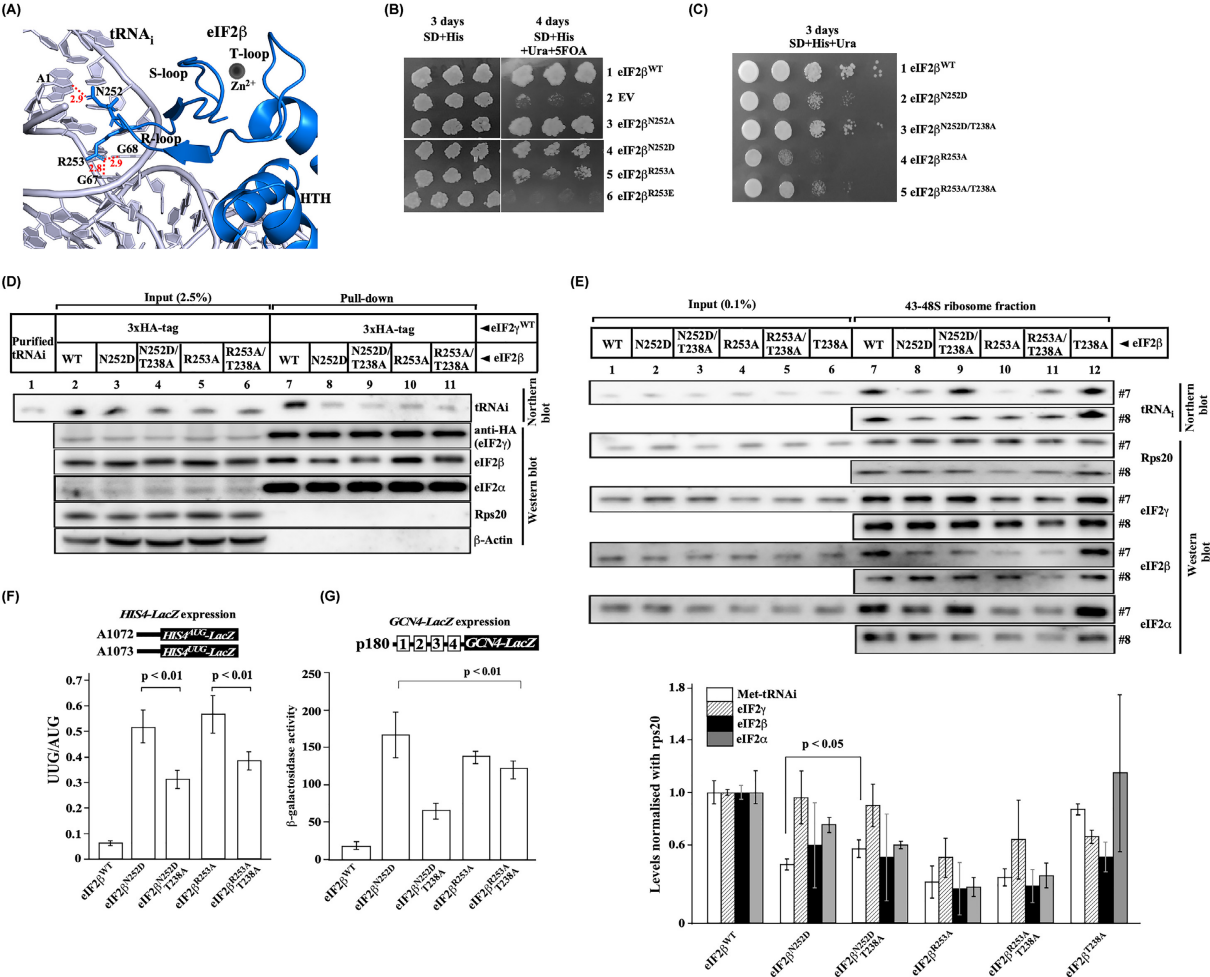
Analysis of eIF2β ZBD residues involved in the Met-tRNA_i_^Met^ binding (**A**) Schematic of eIF2β and Met-tRNA_i_ interaction. The cryoEM structure (6FYX) showing eIF2β (blue) and Met-tRNA_i_ (grey), as per Figure 1B. The eIF2β residues N252 and R253 are represented as sticks and show interactions with the Met-tRNAi residues A1, G67, and G68, indicated in red dotted lines. The distance indicated between these residues is in Å. (**B,C**) Growth analysis. Yeast strain, YP912 (*gcd11Δ, sui3Δ*) carrying YCplac22_HA_GCD11 (A1404) and derivatives of eIF2β^WT^ (A1451), eIF2β^N252A^ (A1213), eIF2β^N252D^ (A1214), eIF2β^R253A^ (A1215), eIF2β^R253E^ (A1216) or, empty vector (EV) (A308) were patched on SD plate supplemented with histidine and replica platted on SD plate supplemented with uracil, histidine and 5FOA and incubated for 3–4 days at 30°C for (**B**). Viable cells from the 5-FOA plate were serially diluted and spotted on the SD plate supplemented with uracil and histidine (**C**). (**D**) Yeast cells from (**C**) were subjected to Co-IP followed by Western and Northern blot analysis as described in Figure 2E. (**E**) Analysis of TC on the 43-48S ribosomes. Yeast cells from (**C**) were subjected to 1% HCHO cross-linking, and the amounts of TC on 43-48S ribosomes were performed as described in Figure 2F. The graph (below) shows the densitometric quantitation of the TC on 40S ribosome from at least three biological replicates. (**F**) Analysis of *HIS4-lacZ* expression. Yeast cells from (**C**) were transformed with either pA1072 (GAPDH_prom__His4^AUG^_lacZ) or pA1073 (GAPDH_prom__His4^UUG^_lacZ) plasmids, and the β-galactosidase assay was performed as described for Figure 2C. (**G**) Analysis of *GCN4-lacZ* expression. Yeast cells from (**C**) were transformed with *GCN4-lacZ* construct (p180), and the β-galactosidase assay was performed as described in Figure 2C. Statistical differences were determined by one-way ANOVA analysis. The error bar shows the standard deviation obtained from at least three biological replicates.

The structure of the eIF2β suggests that the R-loop (resides 245-256) of the ZBD interacts with the Met-tRNA_i_^Met^, and the residues Asn252 and Arg253 may be critical for the Met-tRNA_i_^Met^ binding (Figure 3A). To test this, we mutated Asn252 to Ala or Asp, while Arg253 was mutated to Ala or Glu. After the eviction of the eIF2β^WT^ plasmid, the eIF2β^N252A^ mutation showed no growth defect, whereas the eIF2β^N252D^ mutation caused a growth defect. The eIF2β^R253A^ mutation caused severe growth defects, whereas the eIF2β^R253E^ mutation was lethal, suggesting that the R-loop region is necessary for the Met-tRNA_i_^Met^ binding (Figure 3B). Since the eIF2β^T238A^ (T-loop) suppressor mutation showed higher affinity for the Met-tRNA_i_^Met^ binding (Figure 2E), we reasoned that combining this suppressor mutation with the R-loop mutations should rescue its slow growth defect. Consistently, the slow growth phenotype of the eIF2β^N252D^ and eIF2β^R253A^ mutation was partially suppressed when combined with the T-loop eIF2β^T238A^ mutation (Figure 3C), suggesting that the R-loop and T-loop coordinate to interact with the Met-tRNA_i_^Met^.

To understand the nature of TC formation in the eIF2β^N252D^ and eIF2β^R253A^ mutants, we performed an in-vivo co-immunoprecipitation assay as describes in the previous section. The eIF2β^N252D^ and eIF2β^R253A^ mutations showed the Met-tRNA_i_^Met^ binding defect, suggesting destabilization of the TC (Figure 3D). Consistent with its growth defect, the eIF2β^N252D^ mutant showed a lower amount of the TC binding to the 43-48S ribosomal complex, whereas the eIF2β^N252D/T238A^ double mutation partially rescued the growth defect and improved the Met-tRNA_i_^Met^ levels on the 43-48S ribosomal complex (Figure 3E). However, the eIF2β^R253A^ and the eIF2β^R253A/T238A^ double mutant exacerbate the TC binding to the 43-48S ribosomal complex compared with the eIF2β^N252D^ mutation, which is consistent with its severe slow growth phenotype (Figure 3E).

The eIF2β^N252D^ and eIF2β^R253A^ mutations showed Sui¯ phenotype that can be partially suppressed when combined with the eIF2β^T238A^ mutation (Figure 3F), suggesting that the T-loop eIF2β^T238A^ substitution influences the R-loop residues to alter the Met-tRNA_i_^Met^ binding conformation to partially rescue the Sui¯ phenotype. Consistent with its Met-tRNA_i_^Met^ binding defects, the eIF2β^N252D^ and eIF2β^R253A^ mutations showed Gcd¯ phenotype. Interestingly, the eIF2β^N252D/T238A^ mutation partially rescued the Gcd¯ phenotype of the original eIF2β^N252D^ mutation; however, the eIF2β^R253A/T238A^ did not rescue the Gcd¯ phenotype of the original eIF2β^R253A^ mutation (Figure 3G).

Additionally, we tested if the eIF2β^T238A^ mutation suppresses the defects associated with the previously reported eIF2β^F217A/Q221A^ mutations in the HTH region [3,26]. Combining the eIF2β^T238A^ mutation with the eIF2β^F217A/Q221A^ mutations partially suppressed the Sui¯ and the Gcd¯ phenotype without rescuing its slow growth defect (Supplementary Figure S4). Taken together, these results suggest that the T-loop eIF2β^T238A^ substitution plays an important role not only in the rescue of the eIF2β^S264Y^ binding defect but also influences the eIF2β R-loop and HTH region in partially rescuing the defect in the start codon recognition.

### The eIF2β^T238A^ mutation suppresses the defect associated with the eIF2γ^N135D^ switch-I mutation but not the eIF2γ^V281K^ MEHMO mutation

**Figure 4:**
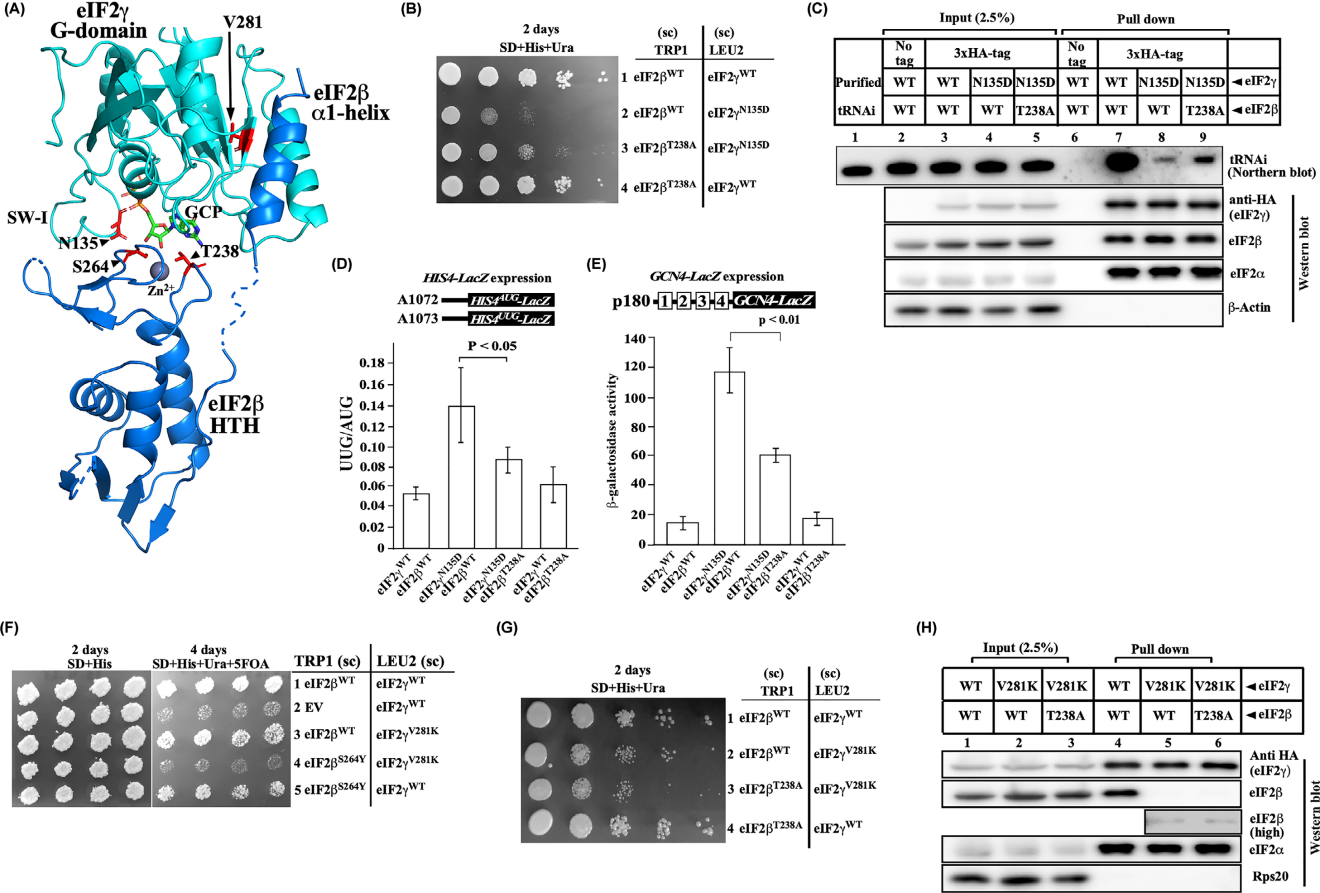
The eIF2β^T238A^ mutation suppresses defects associated with the eIF2γ^N135D^ SW-I mutation (**A**) Schematic of eIF2β and eIF2γ interaction. The cryoEM structure of partial yeast 48S PIC (6FYX) showing eIF2β (blue), eIF2γ (cyan), Zn^2+^ ion (sphere; grey) and a GCP molecule. Residues that were mutated in the present study are shown in red color sticks. (**B**) Growth analysis. Yeast strain YP912 (*gcd11Δ, sui3Δ*) carrying 3xHA-tagged eIF2γ^WT^ (A1) or, eIF2γ^N135D^ (A2) were transformed with eIF2β^WT^ (A1452) or, eIF2β^T238A^ (A1272) plasmid constructs. The cells were grown overnight, serially diluted, spotted on SD plate supplemented with uracil and histidine, and incubated at 30°C for 2 days. (**C**) Co-Immunoprecipitation assay. Yeast cells from (**B**) were subjected to Co-IP followed by Western and Northern blot as described in Figure 2E. (**D**) Analysis of *HIS4-lacZ* expression. Yeast cells from (**A**) were transformed with either GAPDH_prom__His4^AUG^_lacZ (A1072) or GAPDH_prom__His4^UUG^_lacZ (A1073) plasmids, and the β-galactosidase assay was performed as described for Figure 2C. (**E**) Analysis of *GCN4-lacZ* expression. Yeast cells from (**B**) were transformed with *GCN4-lacZ* construct (p180), and the β-galactosidase assay was performed as described in Figure 2D. (**F**) Growth analysis. Yeast strain YP912 (*gcd11Δ, sui3Δ*) carrying empty vector (EV), eIF2β^WT^ (pA1452) or, eIF2β^S264Y^ (pA890) were transformed with eIF2γ^WT^ (pA343) or, eIF2γ^V281K^ (pA1421) or, eIF2γ^N135D^ (pA57) plasmid constructs. The transformant colonies were patched on an SD plate supplemented with histidine, replica plated on an SD plate supplemented with uracil, histidine and 5-FOA, and incubated for 2–4 days at 30°C. (**G**) Growth analysis. Yeast strain YP912 (*gcd11Δ, sui3Δ*) carrying eIF2β^WT^ (A1452)/eIF2γ^WT^ (A1), eIF2β^WT^ (A1452)/eIF2γ^V281K^ (A1426), eIF2β^T238A^ (A1272)/eIF2γ^V281K^ (A1426), or eIF2β^T238A^ (A1272)/ eIF2γ^WT^ (A1) plasmid constructs were grown overnight, serially diluted, and spotted on SD plate supplemented with uracil and histidine and incubated at 30°C for 2 days. (**H**) Co-Immunoprecipitation assay. Yeast cells from (**G**) were subjected to Co-IP followed by Western blot, as described in Figure 2E. A higher exposure (high) of the eIF2β blot for lanes 5 and 6 is shown. Statistical differences were determined by one-way ANOVA analysis. The error bar shows the standard deviation obtained from at least three biological replicates.

The eIF2γ SW-I and SW-II regions play an important role in the effector function of the Met-tRNA_i_^Met^ binding and GTPase activity [4,17,24]. The SW-I region is proposed to adopt a different conformation in the GTP bound conditions to enable effector function compared with the GDP bound state [40]. Structural data suggests that the eIF2γ SW-I residue Arg127 interacts with the eIF2β Thr265 residue (S-loop) of the ZBD in the TC (Figures 1B and 4A) [5]. It is possible that the eIF2β-ZBD may be playing an important role in stabilizing the GTP bound SW-I conformation to enable the Met-tRNA_i_^Met^ binding. Previous studies on the eIF2γ^N135D^ SW-I mutation show defects in the Met-tRNA_i_^Met^ binding, Slg¯, Sui¯, and Gcd¯ phenotype [17]. To check if the eIF2β-ZBD plays an important role in the stabilization of the GTP bound SW-I conformation, we expressed the eIF2γ^N135D^ and the eIF2β^T238A^ mutation together in the YP912 (*gcd11Δ, sui3Δ*) yeast strain. Consistent with our postulation, the eIF2γ^N135D^ SW-I mutant’s severe growth defect was partially rescued by the eIF2β^T238A^ mutation (Figure 4B), suggesting that the eIF2β^T238A^ mutation may alter the eIF2β-ZBD conformation that influences the eIF2γ^N135D^ mutant SW-I region to adopt a favourable conformation to bind the Met-tRNA_i_^Met^. To confirm the better Met-tRNA_i_^Met^ binding activity in the double mutant, we performed an *in vivo* co-immunoprecipitation assay. Consistent with the previous report, the eIF2γ^N135D^ mutation showed severe defects in the Met-tRNA_i_^Met^ binding (Figure 4C, lane 8) [17]. However, co-expression of the eIF2γ^N135D^ and eIF2β^T238A^ mutation partially rescued the Met-tRNA_i_^Met^ binding defect (Figure 4C, lane 9). Consistently, the eIF2γ^N135D^ mutant's Sui¯ and Gcd¯ phenotype was partially suppressed by the eIF2β^T238A^ mutation, indicating that the altered Met-tRNA_i_^Met^ binding affinity adversely affects TC formation (Figure 4D,E).

**Figure 5:**
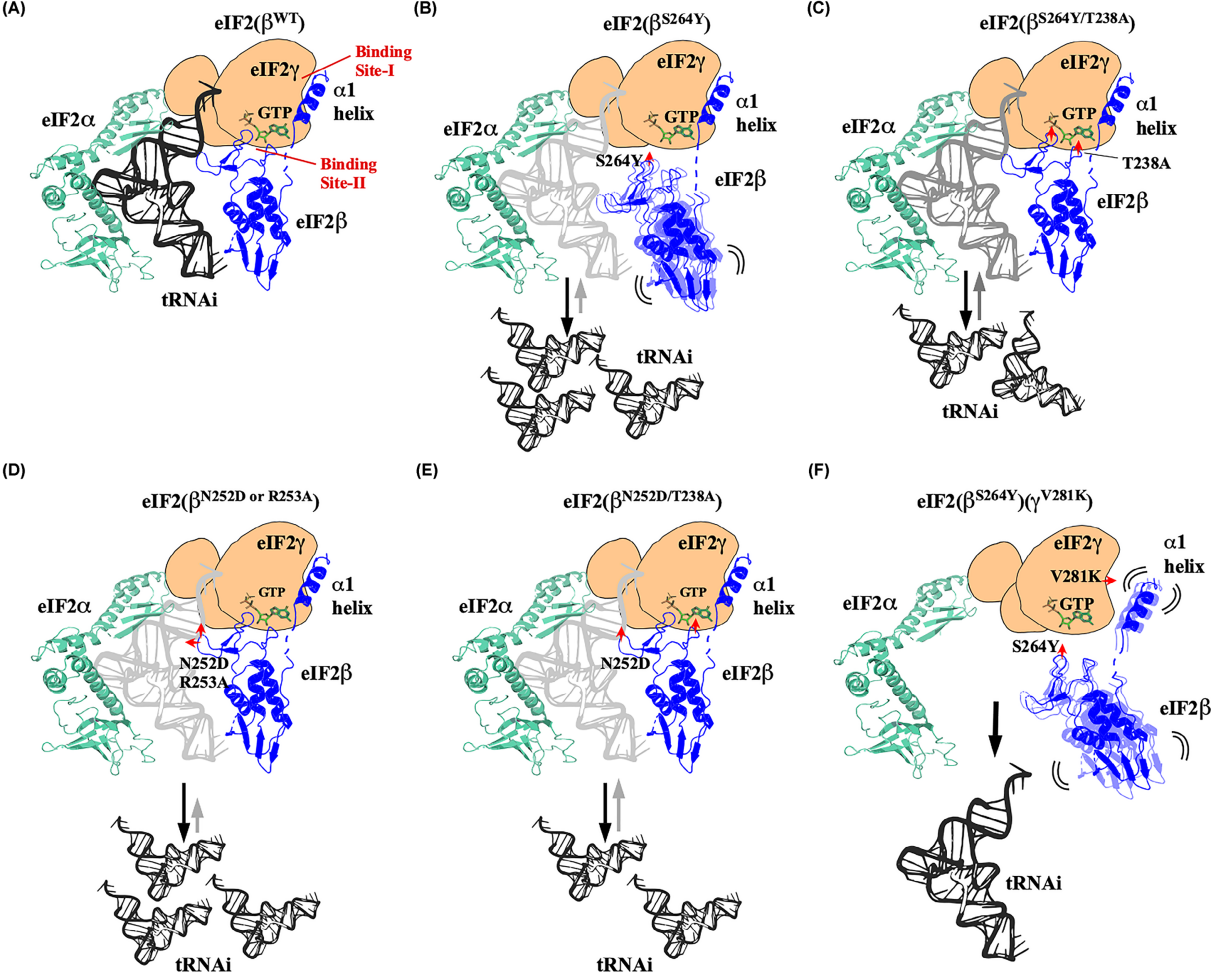
Models depicting the locations of eIF2 mutations and their impacts on Met-tRNA_i_^Met^ and eIF2β-γ binding eIF2γ (light orange), eIF2β (blue), eIF2α (teal), GTP (green), and Met-tRNA_i_^Met^ (black). (**A**) The WT eIF2 complex showing interactions of eIF2α, eIF2β, and Met-tRNA_i_^Met^ with the eIF2γ subunit. The eIF2β α1-helix and ZBD interact with the eIF2γ G-domain at site-I and site-II (guanine nucleotide binding interphase), respectively. (**B**) The eIF2β^S264Y^ ZBD (S-loop) mutation (red arrowhead) disrupts the interaction with the eIF2γ G-domain at site-II. However, the α1-helix remains anchored at the site-I region, making the eIF2β C-terminal domain highly mobile, causing dissociation of the Met-tRNA_i_^Met^ (grey) from the TC. (**C**) The suppressor mutation eIF2β^S264Y/T238A^ in the T-loop (red arrowhead) restores the eIF2β ZBD binding to the site-II. However, the Met-tRNA_i_^Met^ binding defect is partially rescued. (**D**) The eIF2β^N252D^ and eIF2β^R253A^ mutations (red arrowhead) in the R-loop disrupt the interaction with the Met-tRNA_i_^Met^, causing it to dissociate from the TC. (**E**) The eIF2β^N252D/T238A^ suppressor mutation (red arrowheads) partially restores the Met-tRNA_i_^Met^ binding. (**F**) The eIF2β^S264Y^ and eIF2γ^V281K^ mutation (red arrowheads) disrupt the interaction of eIF2β with the eIF2γ subunit both at the site-I and site-II region, preventing eIF2β-γ interaction. In the absence of the eIF2β binding with the eIF2γ subunit, the Met-tRNA_i_^Met^ could not bind to form TC, causing lethality.

MEHMO syndrome is caused by one of the human eIF2γ^I222T^ mutations [41]. Dever and co-workers used yeast as a model system to characterize an eIF2γ^V281K^ mutation (Figure 4A) (corresponding to the human eIF2γ^I222T^ mutation) and showed that the mutation impaired eIF2β binding and enhanced translation initiation from a near-cognate UUG codon [42]. We reasoned that if the eIF2β^T238A^ mutation rescue the eIF2γ-β interaction defect of the eIF2β^S264Y^ mutation, then it could also rescue the eIF2γ-β interaction defect of the eIF2γ^V281K^ MEHMO mutation. However, co-expression of the eIF2γ^V281K^ and eIF2β^T238A^ suppressor mutation did not rescue the growth defect associated with the eIF2γ^V281K^ mutation (Figure 4G, row 3). Moreover, the Co-IP of 3xHA-tagged eIF2γ^V281K^ and eIF2β^T238A^ suppressor mutation did not restore eIF2β-γ interaction (Figure 4H, lane 6). The eIF2β likely has two independent interaction sites on the eIF2γ subunit: The first (site-I), the eIF2β hydrophobic rich α1-helix (residue 127-143) interaction with the eIF2γ surface hydrophobic rich patch residues (276-297) and the second (site-II), the eIF2β-ZBD (T-loop residue 238-240 and S-loop residues 262-265) interaction with GTP binding interface of the eIF2γ subunit (Figure 5A) [3]. Both these interactions could be necessary for the stable eIF2β-γ complex formation and function. However, mutations that disrupt both of these interaction sites should have a catastrophic effect on the TC formation. Consistently, co-expression of the eIF2β^S264Y^ and eIF2γ^V281K^ mutation was lethal in yeast (Figure 4F, row 4 and Figure 5F).

## Discussion

Structural data suggest that the eIF2β-Ser264 residue is part of a zinc-binding domain and interacts via its side chain with the 2′OH and 3′OH groups of the guanine nucleotide’s ribose sugar moiety in the TC. It appears that the eIF2β may be interacting with the core eIF2γ subunit’s guanine nucleotide binding interface via the ZBD, and the eIF2β^S264Y^ mutation may have disrupted this interaction. The yeast two-hybrid and Co-IP experiments confirm the eIF2β^S264Y^ and eIF2γ interaction defect (Figure 1D,E). Biochemical characterization of the eIF2β^S264Y^ mutation and the suppressor mutation revealed no significant changes in the guanine nucleotide binding affinity. However, the eIF2β^T238A^ mutation partially suppresses the Sui¯ and Gcd¯ phenotype of the eIF2β^S264Y^ mutation, suggesting the importance of the T-loop residue in the TC formation (Figure 2C,D). The Co-IP assay and the analysis of the initiation factors associated with the 40S ribosomal subunit confirm the eIF2β and Met-tRNA_i_^Met^ binding defect in the eIF2β^S264Y^ mutation. The eIF2β^S264Y^ mutation may have disrupted the S-loop conformation, leading to the eIF2β interaction defect. In the absence of the stable eIF2β association with the eIF2γ subunit’s guanine nucleotide binding interface, the Met-tRNA_i_^Met^ frequently falls from the eIF2γ subunit, thus lowering the TC levels (Figure 5B). However, whereas the eIF2β^T238A^ suppressor mutation in the T-loop fully rescued the eIF2β binding defect of the eIF2β^S264Y^ mutation, the partial Met-tRNA_i_^Met^ binding defect persists, probably because of the continuous interference of the Ser264Tyr mutant residue with the Met-tRNA_i_^Met^ binding pocket, thus showing the partial Sui¯ and Gcd¯ phenotype (Figures 2E,F and 5C). Therefore, the eIF2β subunit may be contributing to the Met-tRNA_i_^Met^ binding via R-loop as well as the polar interaction of S-loop's Thr265 residue with the eIF2γ subunit’s Arg127 in the SW-I region (Figure 1B). The T-loop eIF2β^T238A^ mutation could likely influence the R-loop and S-loop conformation for the Met-tRNA_i_^Met^ binding. Previous studies on the eIF2γ^N135D^ mutation in the SW-I region show Met-tRNA_i_^Met^ binding defect and an attendant Sui¯ and Gcd¯ phenotype [17]. However, when the eIF2β^T238A^ mutation was combined with the eIF2γ^N135D^ SW-I mutation, it partially suppresses its Slg¯, Sui¯, and Gcd¯ phenotype (Figure 4A–D). This reinforces the notion that the eIF2β-ZBD could perturb the eIF2γ SW-I conformation to maintain the geometry of the Met-tRNA_i_^Met^ binding pocket.

The R-loop is projected into the Met-tRNA_i_^Met^ acceptor arm. The cryo-EM structure data suggest that this R-loop’s Asn252 and Arg253 residue interact with the A1 and G67/G68 nucleotide residue of the Met-tRNA_i_^Met^ acceptor arm, respectively (Figure 3A) [5]. The mutation of Asn252 to the negatively charged aspartic acid may disrupt interaction with the A1 nucleotide, causing the Met-tRNA_i_^Met^ binding defect (Figure 5D), which is consistent with the low levels of Met-tRNA_i_^Met^ on the 40S ribosomal subunit. Whereas the more severe Arg253 to Ala mutation could disrupt the two-hydrogen bond interaction with the G67/G68 Met-tRNA_i_^Met^ nucleotide residues and showed severe slow growth phenotype, the Arg253 to negatively charged glutamic acid substitution was lethal. Remarkably, the T-loop eIF2β^T238A^ mutation partially suppresses the Met-tRNA_i_^Met^ binding defect, Sui¯, and Gcd¯ phenotype of the R-loop eIF2β^N252D^ mutation. We propose that the eIF2β zinc-binding domain's S-loop and T-loop coordinate with the Zn^2+^ ion to facilitate the eIF2β subunit's interaction with the guanine nucleotide-binding interface of the eIF2γ G-domain and help to position HTH domain and the R-loop to interact with the Met-tRNA_i_^Met^ via Asn252 and Arg253 residue. However, the eIF2β^T238A^ mutation could perturb these loop’s conformation and subtly orient the S-loop and R-loop conformation to enhance the interaction and orientation of the Met-tRNA_i_^Met^ binding with the eIF2 complex (Figure 5E). This could be the reason why the eIF2β^T238A^ mutation showed a strong affinity (∼12.5-fold) for the Met-tRNA_i_^Met^ binding to the eIF2 complex (Figure 2E, lane 16). However, this augmented Met-tRNA_i_^Met^ binding affinity in the eIF2β^T238A^ mutant may be only restricted to the TC complex formation level. Once the TC is loaded onto the 40S ribosomal subunit, the Met-tRNA_i_^Met^ binding affinity of the eIF2β^T238A^ mutant to the 43-48S complex is comparable to the WT levels (Figure 2E, lane 16 and Figure 2F).

Overall, the partial rescue of the Sui¯ and Gcd¯ phenotype of eIF2β S-loop (S264Y), R-loop (N252D), HTH (F217/Q221A) and the eIF2γ SW-I (N135D) mutations by the eIF2β T-loop (T238A) mutation reinforce the notion that the eIF2β-ZBD modulates the Met-tRNA_i_^Met^ binding affinity through the guanine nucleotide-binding interface. It not only moderately restores the Met-tRNA_i_^Met^ binding to alleviate the Gcd¯ phenotype but also prevents premature delivery of the Met-tRNA_i_^Met^ in the P_IN_ conformation to partially rescue the Sui¯ phenotype.

Castilho and coworkers have previously established that the eIF2β region from residue 128-159 is sufficient to interact with the eIF2γ subunit. They also demonstrated that the eIF2β^Y131A/S132A^ double mutation causes an eIF2γ subunit binding defect. Moreover, combining the eIF2β^S264Y^ mutation with eIF2β^Y131A/S132A^ mutation causes lethality [32]. The cryo-EM data suggest that the eIF2β hydrophobic amino acid-rich α1-helix interacts with the eIF2γ-G domain surface hydrophobic rich patch residues (276-297) [3]. Interestingly, the cryo-EM structure of partial yeast 48S complex containing eIF2β^S264Y^ mutation subunit shows only α1-helix interacting with the eIF2γ-G domain; however, the eIF2β-ZBD and HTH domain is not resolved in this structure [15], possibly high mobility of these domains (Figure 5B). These data and our findings indicate that the eIF2β-ZBD interacts with the eIF2γ subunit via the guanine nucleotide-binding interface. Moreover, the inability of the eIF2β^T238A^ mutation to suppress the growth defect of the eIF2γ^V281K^ G-domain mutation and the co-expression of the eIF2β^S264Y^ and eIF2γ^V281K^ mutation causing synthetic lethality suggest that the eIF2β may have two independent interaction sites on the eIF2γ subunit (Figures 4F–H and 5F). We propose the eIF2β α1-helix interaction with the eIF2γ G-domain could be a primary binding site (site-I), whereas a slightly flexible eIF2β-ZBD interaction with the guanine nucleotide-binding interface may be acting as a secondary binding site (site-II). Both these interactions of eIF2β with the eIF2γ subunit are critical for binding the Met-tRNA_i_^Met^ to the eIF2 complex (Figure 5A). Thus, the eIF2β subunit anchors on the eIF2γ G-domain via its α1-helix, whereas the flexible secondary binding between the eIF2β-ZBD and eIF2γ guanine nucleotide-binding interface may allow eIF2β subunit movement to load and stabilize the Met-tRNA_i_^Met^ during the TC formation and Met-tRNA_i_^Met^ unloading during delivery to the P-site of 40S ribosome.

## Materials and methods

### Growth media

1. Synthetic dextrose (SD): 0.17% yeast nitrogen base without NH_4_(SO_4_), 0.5% NH_4_(SO_4_)_2_, 2% glucose.
2. Synthetic complete dropout (SCD): 0.17% yeast nitrogen base without NH_4_(SO_4_), 0.5% NH_4_(SO_4_)_2_, 2% glucose supplemented with adenine, alanine, arginine, asparagine, aspartate, cysteine, glutamine, glutamate, glycine, inositol, isoleucine, lysine, methionine, p-amino benzoic acid, phenyl alanine, proline, serine, threonine, tyrosine, and valine (2 mg/ml each).

### Preparation of yeast strain (Table 1)

The oligonucleotides (Table 3) oPA1117/oPA1118 carrying *SUI3* ORF specific flanking sequence was used to PCR amplify 2.5 kb *LoxP-LEU2-LoxP* cassette from the pUG73 (B4034) plasmid template and transformed into the yeast strain YP824 harbouring *SUI3_GCD11*/URA3 (A1118) plasmid to delete the chromosomal *SUI3* gene by homologous recombination method. The oligonucleotide oPA887 and oPA160 binds to the *LEU2* cassette and the *SUI3* 3′ UTR region, respectively and gives 1 kb PCR amplification from the genome of this intermediate strain which confirmed the insertion of the *LoxP-LEU2-LoxP* cassette and removal of the *SUI3* ORF. The Cre recombinase enzyme expression from the plasmid YCplac22_Gal_Cre [43] removed the *LoxP-LEU2-LoxP* DNA sequence to generate yeast strain YP896 [43]. The oligonucleotides oPA929/oPA930 carrying *GCD11* ORF specific flanking sequence were used to PCR amplify 2.5 kb *LoxP-LEU2-LoxP* cassette from the pUG73 plasmid template and transformed into the yeast strain YP896 to delete the chromosomal *GCD11* gene by homologous recombination method. The oligonucleotide oPA887 and oPA390 binds to the *LEU2* cassette and the *GCD11* 3′ UTR region, respectively and amplify 1 kb PCR product from the genome of this intermediate strain which confirmed the insertion of the *LoxP-LEU2-LoxP* cassette and removal of the *GCD11* ORF from the genome. The Cre recombinase enzyme expression from the plasmid YCplac22_Gal_Cre further removed the *LoxP-LEU2-LoxP* DNA sequence to generate a final yeast strain YP912. Table 1.

**Table 1:** List of yeast strains used in the present study

Sr. No.	Strain	Genotype	Reference
1	YP824	*MATα leu2-3, -112, ura3-52, trp1-63Δ, GCN2+, Gal2+ his4Δ::KanMx6*	[28]
2	YP896	*MATα ura3-52, leu2-3,-112 trp1-63*Δ *his4::KanMx6 sui3::LoxP GAL2+ p[GCD11-SUI3,URA3]*	This study
3	YP912	*MATα ura3-52 leu2-3,-112 trp1-63*Δ *his4::KanMx6 sui3::LoxP gcd11::LoxP GAL2+ p[GCD11-SUI3,URA3]*	This study
4	YP920	*MATa his3-*Δ*1 ura3-*Δ*0 leu2*Δ*0 trp1-1 met15-*Δ*0 sui2*Δ::*hisG sui3*Δ::*KanMX4 gcd11*Δ::*NAT gcn2*Δ::*hisG pep4::HIS3 p1780[SUI2, SUI3, GCD11, URA3]*	[22]
5	YP930	*MATa trp1-901 leu2-3 leu2-112 ura3-52 his3-200 ade2-201 gal4-Δ gal80-ΔLYS2::GAL1UAS-GAL1TATA-HIS3, GAL2UAS-GAL2TATA-ADE2 URA3::MEL1UAS-MEL1TATA-lacZ*	[44]

### Cloning and plasmid preparations (Table 2)

The oligonucleotide pair oPA160/oPA161 (Table 3) was used to PCR amplify *SUI3* gene from the yeast chromosome, the PCR product and the vectors YCplac111_EV (A308) and YCplac22_EV (A823) were digested with the BamHI-SalI restriction endonuclease (RE) and ligated to generate recombinant plasmid YCplac111_SUI3 (A1451) and YCplac22_SUI3 (A1452), respectively. The YCplac111-SUI3-S264Y (A1446) and YCplac33-SUI3-S264Y (A1084) construct were generated by subcloning a BamHI-SalI digested 1.9 kb fragment from the pRS313-SUI3-S264Y (A479) plasmid and ligated into the BamHI-SalI digested YCplac111-EV (A308) and YCplac33-EV (A309) vector, respectively. Tables 2 and 3.

**Table 2:** List of plasmids used in the present study

Sr No	Plasmid No.	Plasmid name	Plasmid type	Reference
1	A1	YCplac111_HA_GCD11	Single Copy	This study
2	A2	YCplac111_HA-GCD11-N135D	Single Copy	This study
3	A57	YCplac111_GCD11-N135D	Single Copy	[17]
4	A200	YCplac111_SUI3_prom_+HA	Single Copy	This study
5	A202	YCplac111_HA_SUI3	Single Copy	This study
6	A308	YCplac111_EV	Single Copy	[45]
7	A309	YCplac33_EV	Single Copy	[45]
8	A325	YCplac111_GCD11_prom_	Single Copy	This study
9	A326	YCplac111_GCD11_prom_+HA	Single Copy	This study
10	A343	YCplac111_GCD11	Single Copy	[17]
11	A479	pRS313_SUI3-S264Y	Low Copy	This study
12	p180	YCplac33_GCN4_lacZ	Single Copy	[46]
13	A591	pRS425_EV	High Copy	[47]
14	A643	pDEST22_AD (Empty Vector)	High Copy	Invitrogen
15	A644	pEXP32-Krev1		Invitrogen
16	A823	YCplac22_EV	Single Copy	[45]
17	A839	YCplac33_His4^AUG^	Single Copy	[48]
18	A840	YCplac33_His4^UUG^	Single Copy	[48]
19	A890	YCplac22_Sui3-S264Y	Single Copy	[28]
20	A1072	YCplac33_GAPDH_prom__His4^AUG^_lacZ	Single Copy	[48]
21	A1073	YCplac33_GAPDH_prom__His4^UUG^_lacZ	Single Copy	[48]
22	A1084	YCplac33_Sui3-2	Single Copy	This study
23	A1095	YCplac111_GCD11, SUI3	Single Copy	This study
24	A1118	YCplac33_GCD11, SUI3	Single Copy	This study
25	A1213	YCplac111_SUI3-N252A	Single Copy	This study
26	A1214	YCplac111_SUI3-N252D	Single Copy	This study
27	A1215	YCplac111_SUI3-R253A	Single Copy	This study
28	A1216	YCplac111_SUI3-R253E	Single Copy	This study
29	A1236	pDEST32_mod_ (Empty Vector)	High Copy	This study
30	A1237	pDEST32_mod__SUI3	High Copy	This study
31	A1238	pDEST32_mod__SUI3-S264Y	High Copy	This study
32	A1242	pDEST22_GCD11	High Copy	This study
33	A1259	YCplac111_SUI3-S264Y/T238A	Single Copy	This study
34	A1260	YCplac111_SUI3-T238A	Single Copy	This study
35	A1266	pRS425_GCD11, SUI3, SUI2	High Copy	This study
36	A1272	YCplac22_SUI3-T238A	Single Copy	This study
37	A1394	YCplac111_HA_SUI3-T238A	Single Copy	This study
38	A1404	YCplac22_HA_GCD11	Single Copy	This study
39	A1413	pRS425_GCD11, SUI3-S264Y/T238A, SUI2	High Copy	This study
40	A1414	pRS425_GCD11, SUI3-T238A, SUI2	High Copy	This study
41	A1421	YCplac111_GCD11-V281K	Single Copy	This study
42	A1426	YCplac111_3xHAGCD11-V281K	Single Copy	This study
43	A1446	YCplac111_SUI3-S264Y	Single Copy	This study
44	A1451	YCplac111_SUI3	Single Copy	This study
45	A1452	YCplac22_SUI3	Single Copy	This study
46	A1453	YCplac22_GCD11	Single Copy	This study
47	A1488	YCplac111_SUI3_prom_	Single Copy	This study
48	A1490	YCplac111_SUI3-F217A/Q221A	Single Copy	This study
49	A1492	YCplac111_SUI3-F217A/Q221A/T238A	Single Copy	This study
50	A1493	YCplac111_SUI3-N252D/T238A	Single Copy	This study
51	A1494	YCplac111_SUI3-R253A/T238A	Single Copy	This study
52	A1507	pRS425_GCD11, SUI3-S264Y, SUI2	High Copy	This study
53	B4034	pUG73		[43]

**Table 3:** List of oligonucleotides used in the present study

Sr No	Oligonucleotide Name	Sequence (5′ → 3′)
1	oPA160	CGAGGATCCTGGGCGTCGTTGAATGGC
2	oPA161	CGCAGTCGACCCTGTCCTTGGGAAGTAAAC
3	oPA321	AGCGGATCCAGCGTAATCTGGAACGTCATATGGATATCCTGCATAGTCCGGGACGTCATACGGATAGCCCGCATAGTCAGGAACATCGTATGGGTAGGACATCTCGTGCGTGCTTATTATATGACTGG
4	oPA390	CAGAGAGCTCCAGATCCAACCGCGGGAAGTGGC
5	oPA391	CGCGGATCCGACTTACAAGACCAAGAACCTAGC
6	oPA393	AGCGGATCCGTGATGGTGATGGTGATGGTGATGTCCGGACATGTCTACCTCTAATGCGCGATG
7	oPA887	AGTTATCCTTGGATTTGG
8	oPA929	TCGCGCATTAGAGGTAGACATGAGTGACTTACAAGACCACAGCTGAAGCTTCGTACGC
9	oPA930	TGGTTTTATTGGTTCCTTAAGCGATGGGTTCCAATGCATAGGCCACTAGTGGATCTG
10	oPA1092	GACAGGATCCTCCTCCGATTTAGCTGCTG
11	oPA1117	GTCATATAATAAGCACGCACGAGATGTCCTCCGATTTACAGCTGAAGCTTCGTACGC
12	oPA1118	GTAAAGCACCAACATCACATTCTCCTTCTCTTAGCATAGGCCACTAGTGGATCTG
13	oPA1282	GTACGCGGCCGCTCTCCATGTACAAACCACCGATAAG
14	oPA1283	GTCAACTAGTTGGGCGTCGTTGAATGGC
15	oPA1284	CAGTCCCGGGCCTGTCCTTGGGAAGTAAAC
16	oPA1285	CTAGCCCGGGCCTGAATTCAGTTCTACTGGGATGA
17	oPA1286	GTCACTCGAGCAATGATTTAAATGCAATTCCGAAGGA
18	oPA1299	GAGAGAACAGTCAGCCAGACTGTTCTTTATGGTCT
19	oPA1300	GAACAGTCTGGCTGACTGTTCTCTCTTCAATTCGGT
20	oPA1301	AGAGAACAGTCAAACGCACTGTTCTTTATGGTCTG
21	oPA1302	AAGAACAGTGCGTTTGACTGTTCTCTCTTCAATTC
22	oPA1307	AGAGAACAGTCAAACGAACTGTTCTTTATGGTCTG
23	oPA1308	AAGAACAGTTCGTTTGACTGTTCTCTCTTCAATTC
24	oPA1309	AGAGAACAGTCAGACAGACTGTTCTTTATGGTCTG
25	oPA1310	GAACAGTCTGTCTGACTGTTCTCTCTTCAATTC
26	oPA1440	CTCCAAGCTTGAAGCAAGCC
27	oPA1441	CACCGAGCTCGCTAGCCCGGGACTAGTCGACGTTTGATTCGACCTCGAC
28	oPA1450	CACGGTCGACTAGTTCCTCCGATTTAGCTGCTG
29	oPA1451	CACCGAGCTCTCACATTCTCCTTCTCTTACC
30	oPA1520	TGTCACTTGTAAAGCGTGTAAGAGTATTAACACCG
31	oPA1521	TACTCTTACACGCTTTACAAGTGACATACTCC
32	oPA1600	CGCAGTCGACGCGGCCGCTCTCCATGTACAAACCACCGATAAG
33	oPA1651	CACCGAGCTCTGGGCGTCGTTGAATGGC
34	oPA1732	GGTTTCGATCCGAGGACATCAGGGTTATGAGCCC
35	oPA1771	CTCCTATTAAGCCAATATCCGCTCAGTTGAAG
36	oPA1772	GGATATTGGCTTAATAGGAGCACCGTCAGC
37	oPA1868	AACTCTCGACCTTCAGA
38	oPA1888	GGGTAAGGCTCAATCCAAAGCTATGGAGAATGTCTTAAG
39	oPAx01	CACCGGATCCAGCGTAATCTGGAACGTCATATGGATATCCTGCATAGTCCGGGACGTCATACGGATAGCCCGCATAGTCAGGAACATCGTATGGGTAGGACATGTCTACCTCTAATGCGCGATG

Introduction of T238A mutation in the *SUI3* gene was accomplished by fusion PCR using oligonucleotide pairs oPA160/oPA1521, oPA1520/oPA161 and YCplac111-SUI3 (A1451) as a template and cloned into the YCplac111-EV (A308) vector at the BamHI-SalI site to generate YCplac111-SUI3-T238A (A1260) construct. To generate the YCplac111-SUI3-T238A/S264Y (A1259) mutant construct, the same oligonucleotide set was used to amplify a 1.9 kb fragment from the pRS313-SUI3-S264Y (A479) plasmid and digested using BamHI-SalI RE and ligated into the YCplac111-EV (A308) vector. The 1.9 kb SUI3-T238A fragment was digested from the YCplac111-SUI3-T238A (A1260) plasmid and subcloned into the YCplac22-EV (A823) vector at the BamHI-SalI site to generate YCplac22-SUI3-T238A (A1272) construct.

The *SUI3* mutations N252A and N252D were introduced by fusion PCR (1.9 kb) using oligonucleotide pairs oPA160/oPA1300, oPA1299/oPA161 and oPA160/oPA1310, oPA1309/oPA161, respectively using YCplac111-SUI3 (A1451) template. The PCR amplified products were digested using BamHI-SalI RE and ligated into the YCplac111-EV (A308) vector to generate YCplac111-SUI3-N252A (A1213) and YCplac111-SUI3-N252D (A1214) constructs. The *SUI3* mutations R253A and R253E were introduced by fusion PCR using oligonucleotide pairs oPA160/oPA1302, oPA1301/oPA161 and oPA160/oPA1308, oPA1307/oPA161, respectively and YCplac111-SUI3 (A1451) as a template. The PCR amplified products were digested using BamHI-SalI RE and ligated into YCplac111-EV (A308) vector to generate YCplac111-SUI3-R253A (A1215) and YCplac111-SUI3-R253E (A1216) constructs.

The site-directed mutations SUI3-N252D/T238A and SUI3-R253A/T238A were generated by fusion PCR amplification using the oligonucleotide pair oPA160/oPA1521, oPA161/oPA1520 from YCplac111-SUI3-N252D (A1214) and YCplac111-SUI3-R253A (A1215) template, respectively. The 1.9 kb PCR amplified product was digested using BamHI-SalI RE and ligated into the YCplac111-EV (A308) vector to generate YCplac111-SUI3-N252D/T238A (A1493) and YCplac111-SUI3-R253A/T238A (A1494) constructs, respectively.

The 1.9 kb *SUI3* gene was amplified from the YCplac111-SUI3 (A1451) plasmid using oligonucleotide pair oPA1077/oPA161 and digested using SphI-SalI RE. The digested product was ligated into the YCplac111-GCD11 (A343) plasmid to generate YCplac111-GCD11-SUI3 (A1095) plasmid. The 4.4 kb GCD11-SUI3 fragment was digested from the YCplac111_GCD11, SUI3 (A1095) plasmid and subcloned into YCplac33 (A309) at the SphI-SacI site to generate YCplac33-GCD11-SUI3 (A1118) plasmid. To add N-terminal 3x-HA tag at the eIF2β subunit, a 0.7 kb SUI3_prom+3xHA PCR product was amplified using oligonucleotide pair oPA1651/oPA321 and YCplac111-SUI3 (A1451) as a template and ligated into the YCplac111 (A308) vector at the SacI-BamHI site to generate YCplac111-SUI3_prom_+3xHA (A200) intermediate construct. The 1.1 kb *SUI3* ORF+terminator region was PCR amplified using oligonucleotides oPA1092/oPA161 and ligated into the intermediate construct A200 at the BamHI-SalI site to generate YCplac111-3xHA-SUI3 (A202) plasmid. The 1.1 kb SUI3-T238A fragment was PCR amplified using oligonucleotides oPA1092/oPA161 and YCplac111-SUI3-T238A (A1260) as a template and ligated at the BamHI-SalI site of the intermediate construct to generate the YCplac111-3xHA-SUI3-T238A (A1394) plasmid.

The F217A/Q221A mutation was introduced into the *SUI3* gene by PCR amplifying the 1.1 kb fragment using oligonucleotide pair oPA1092/oPA1889, oPA161/oPA1888, and YCplac111-SUI3 (A1451) as a template. The amplified product was digested using BamHI-SalI RE and ligated into the YCplac111-SUI3_prom_ (A1488) vector backbone to generate the YCplac111-SUI3-F217A/Q221A construct (A1490). Another construct, YCplac111_SUI3-F217A/Q221A/T238A (A1492), was also generated by a 1.1 kb fusion PCR amplified product from YCplac111-SUI3-T238A (A1260) plasmid template using the same set of oligonucleotide pairs. The BamHI-SalI digested PCR product was ligated into the YCplac111-SUI3_prom_ (A1488) vector backbone.

The YCplac22-3xHA-GCD11 (A1404) and YCplac22-GCD11 (A1453) constructs were generated by digesting ∼2.5 kb *GCD11* and 3xHA-GCD11 product from YCplac111-3xHA-GCD11 (A1) and YCplac111-His_8_-GCD11 (A343) respectively using SacI-SalI RE and subcloned into YCplac22-EV (A823) vector. Using the oPA390/oPA393 oligonucleotide pair, a ∼ 0.6 kb *GCD11* promoter region was PCR amplified from the yeast genome. This PCR product was digested with the SacI-SalI RE and ligated into the YCplac111-EV (A308) plasmid at the SacI-SalI site to generate the YCplac111-GCD11_prom_ (A325) intermediate plasmid construct. Using oPA390/oPAx01 oligonucleotide pair, a ∼0.65 kb *GCD11* promoter and 3xHA tag region was PCR amplified from the yeast genome, digested with the SacI-SalI RE and ligated into the YCplac111-EV (A308) plasmid at the SacI-SalI site to generate the YCplac111-GCD11_prom_ +3xHA (A326) intermediate plasmid construct. YCplac111-3xHA-GCD11 (A1) construct was generated by 2.1 kb PCR amplification of GCD11 ORF+ terminator region from the YCplac111-GCD11 (A343) plasmid using oligonucleotide pair oPA391/oPA1600 and digested PCR product was ligated into the YCplac111-GCD11_prom_+3xHA (A326) vector at the BamHI-SalI site. Using the same set of oligonucleotide pairs, a 2.1 kb PCR amplified product from the YCplac111-GCD11-N135D (A57) plasmid was digested and ligated into the YCplac111-GCD11_prom_ +3xHA (A326) vector at the BamHI-SalI site to generate YCplac111-3xHA-GCD11-N135D (A2) plasmid. The V281K mutation was introduced into the *GCD11* gene by PCR amplification of 2.1 kb product using oligonucleotide pairs oPA391/oPA1772, oPA1771/oPA1600, and YCplac111-His_8_-GCD11 (A343) as a template. The 2.1 kb PCR product was digested using BamHI-SalI RE and cloned into YCplac111_GCD11_prom_ (A325) and YCplac111_GCD11_prom_+HA (A326) vectors to generate YCplac111_GCD11-V281K (A1421) and YCplac111_3xHA-GCD11-V281K (A1426) constructs, respectively.

The 8xHis-tagged *GCD11* gene was PCR amplified from YCplac111-His_8_-GCD11 (A343) plasmid using oligonucleotide pairs oPA390/oPA1282 and digested with the SacI/NotI RE and cloned into the Leu2-HC backbone pRS425 (A591) vector to generate an intermediate vector. The *SUI3* and *SUI2* genes were PCR amplified using oligonucleotide pairs oPA1283/oPA1284 and oPA1285/oPA1286, respectively. The PCR products were digested with SpeI/SmaI and SmaI/XhoI RE and cloned into the above intermediate vector to generate pRS425_GCD11, SUI3, SUI2 (A1266) plasmid. The same set of primers was used to create mutant derivatives of *SUI3* constructs (A1507: S264Y, A1413: S264Y/T238A, A1414: T238A).

The oligonucleotide pair oPA1440/oPA1441 was used to amplify 487 bp DNA Binding Domain (DBD) from the pEXP32-Krev1 (A644) vector. This fragment was cloned into the same vector at the HindIII/SacI site, resulting in the deletion of the Krev1 gene and the introduction of additional restriction sites for further cloning. The resultant plasmid was called pDEST32_mod_ (A1236). The *SUI3* ORF (WT or mutants) were PCR amplified using the oligonucleotide pair oPA1450/oPA1451 from either YCplac111_SUI3 (A1451) or pRS313_SUI3-S264Y (A479) plasmid templates and cloned at the SalI/SacI site, in-frame with the DBD-domain to generate pDEST32_mod__SUI3 (A1237) and pDEST32_mod__SUI3-S264Y (A1238) plasmids, respectively. The *GCD11* ORF was PCR amplified using oligonucleotide pair oPA1448/oPA1449 and YCplac111_GCD11 (A343) as a template, digested with the SalI/SacI RE, and cloned in-frame with the Activation Domain (AD) to generate pDEST22_GCD11 (A1242) construct.

### Screening for the eIF2β^S264Y^ intragenic suppressor mutations

Yeast strain YP896 (*his4Δ, sui3Δ*) was swapped with the eIF2β^S264Y^ mutant *sui3-2*/URA3 plasmid to prepare a strain for the suppressor screening. Separately, YCplac111_SUI3-S264Y (A1446) plasmid was transformed into a hyper-mutagenic XL1-Red *Escherichia coli* strain to generate mutagenic library (sui3-2*/LEU2). The mutagenic plasmid library and the empty vector control were transformed into the above yeast strain and plated on the SD plate supplemented with histidine and tryptophane. Yeast colonies growing faster than the empty vector control transformant were selected for further analysis. These colonies were subjected to the 5-FOA selection to evict the *sui3-2*/URA3 (A1084) plasmid, and the sui3-2*/LEU2 suppressor plasmid was isolated from the resultant mutation was confirmed by DNA sequencing.

### *LacZ* reporter assay to quantitate Sui¯ and Gcd¯ phenotype

The plasmid *HIS4^AUG^-LacZ* (A1072) and *HIS4^UUG^-LacZ* (A1073) were used to quantitate the UUG/AUG ratio, whereas the plasmid *GCN4-lacZ* (p180) was used to quantitate *GCN4* expression as described previously [17,36].

### Yeast two-hybrid assay

A yeast two-hybrid protein interaction assay was performed using the YP930 reporter yeast strain. The wild-type eIF2γ protein was fused with the Activating domain (AD), and the eIF2β^WT^ or eIF2β^S264Y^ mutant protein was fused with the DNA-binding domain (DBD). Yeast cells YP930 were co-transformed with a control plasmid containing DBD and AD empty vector or with the AD-GCD11 construct along with the derivatives of DBD-SUI3 (WT or mutants) constructs. The colonies were grown on the SCD plate supplemented with histidine, uracil and adenine plate. The colonies were patched on the fresh SCD plate supplemented with histidine, uracil and adenine, replica plated or spotted on the SCD plate supplemented with uracil and adenine and SCD plate supplemented with uracil, adenine and 3AT and grown for 2-3 days.

### Co-Immunoprecipitation assay

Yeast cells were grown in 35 ml of SD medium at 30°C to the mid-log phase, harvested by centrifugation at 3000 × ***g*** and resuspended in the lysis buffer (20 mM HEPES, pH 7.5, 100 mM KCl, 5 mM MgCl_2_, 5 mM NaF, 1 mM EDTA, containing one tablet of Roche cocktail protease inhibitor (#05892970001) and 2 μM of each protease inhibitors Aprotinin, Leupeptin, PMSF and Pepstatin). The WCE was prepared by mechanical lysis of cells using glass beads. Monoclonal anti-HA conjugated antibody agarose beads (10 μl) (Sigma, #A2095) were washed 3 times with 1× PBS and resuspended in 10 μl of binding buffer (lysis buffer plus 0.2% Nonidet P-40 and 1% BSA). The yeast WCE (300 μg) was mixed with these beads and incubated in a nutating mixer at 4°C for 1 h. The beads were washed 5 times with the washing buffer (20 mM HEPES pH 7.5, 100 mM KCl, 5 mM MgCl_2_, 5 mM NaF, 1 mM EDTA, 0.2% NP-40). The beads were suspended in 20 μl of the lysis buffer and split into two halves. One half was separated on 12% SDS-PAGE. The resolved proteins were transferred to the PVDF membrane and analysed by the Western blot using anti-eIF2β (BioBharati Life Science), anti-eIF2α (BioBharati Life Science), anti-eIF2γ (BioBharati Life Science), anti-eIF3c (BioBharati Life Science), anti-RPS20 (BioBharati Life Science), anti-actin (Santa Cruz #sc-47778) with mouse monoclonal anti-HA (Sigma, #H9658) antibodies and horseradish peroxidase (HRP) conjugated mouse secondary antibody (Sigma, #A9044). The blot was developed using a SuperSignal kit (Thermo Scientific #34075). The other half of the beads were treated with a Trizol reagent (Ambion #15596026) to extract RNA. The RNA from the aqueous phase was precipitated using two volumes of ice-cold ethanol, 20 µg GlycoBlue, and 1/10th volume of 3M sodium acetate pH 5.0 and incubating at −20°C for 2 h. The RNA pellet was recovered by centrifugation at 13,000xg for 30 min at 4°C and washed with ice-cold 70% ethanol. The RNA pellet was air dried and reconstituted in 8 μl of 2× RNA loading dye (95% formamide, 0.02% SDS, 0.1mM EDTA, 0.01% bromophenol blue, and 0.005% xylene cyanol) and resolved on the 8% Urea-PAGE. The RNA was transferred to the nylon membrane (Cytiva, #10416296) and analysed by the Northern blotting using a biotinylated probe specific against initiator Met-tRNA_i_ (oPA1732) and elongator Met-tRNA_e_ (oPA1868) and developed using Chemiluminescence kit (Thermo # 89880).

### Quantification of blots

The Western and Northern blots were visualized using Vilber Lourmat Fusion Solo S chemiluminescence doc (EvolutionCapt Solo S 17.00 software). Densitometric analysis was performed using NIH ImageJ software. For the Co-IP experiment, different amount of proteins or the Met-tRNAi pull down with the HA-tagged eIF2γ subunit were quantified from the blot by normalizing with the anti-HA band. The quantification of the 43-48S ribosomal fractions was performed by normalizing with the ribosomal protein (RPS20). The significant differences between eIF2 WT and the mutant proteins were performed using one-way ANOVA analysis.

### eIF2 purification

Yeast strain YP920 (*GCD11Δ, SUI2Δ, SUI3Δ*) carrying high copy of *IMT4* and either high copy [pRS425_GCD11, SUI3, SUI2] (A1266), [pRS425_GCD11, SUI3-S264Y/T238A, SUI2] (A1413), [pRS425_GCD11, SUI3-T238A, SUI2] (A1414), or [pRS425_GCD11, SUI3-S264Y, SUI2] (A1507) plasmid were grown in the 6 L SCD+Met liquid culture to A_600_ ∼ 2. The cells were pelleted by centrifugation at 4000 × ***g*** and flash-frozen in the liquid nitrogen. The cells were lysed in using a mixer mill (MM-40) in liquid nitrogen and re-suspended in the lysis buffer [75mM Tris-Cl pH 7.6, 100 mM KCl, 1 mM EDTA, 1 mM EGTA, 100 µM GDP-Mg^2+^, 5 mM NaF, 10 mM β-mercaptoethnol, protease inhibitor cocktail (Roche# 05892791001), and 2 μM of each protease inhibitors Aprotinin, Leupeptin, PMSF and Pepstatin]. The cell lysate was clarified by centrifugation at 13,000xg for 30 min at 4°C and the supernatant was dialysed in the NCLB-20 buffer (20 mM Tris-Cl pH 7.6, 500 mM KCl, 20 mM imidazole, 0.1 mM MgCl_2_, 5 mM NaF, 10 mM β-mercaptoethnol, 10% glycerol and protease inhibitor cocktail). The dialysed sample was incubated with 5 ml Nickel-NTA beads (Qiagen #30230) for 30 min at 4°C and subsequently washed with the NCLB-20 buffer. The 8xHis-tag eIF2 protein or its mutant derivatives were eluted by NCEB-200 buffer (20 mM Tris-Cl pH 7.6, 500 mM KCl, 200 mM imidazole, 0.1 mM MgCl_2_, 10% Glycerol, 5 mM NaF, 10 mM, β- mercaptoethnol, protease inhibitor). The eluted proteins were further dialysed in HSDB buffer (20 mM Tris-Cl pH 7.6, 100 mM KCl, 0.1 mM MgCl_2_, 10% glycerol, 5 mM NaF, 1 mM DTT, protease inhibitor) and the protein was purified sequentially by Heparin and cation exchange column chromatography using 50–1000 mM KCl gradient as described previously [17]. The purified proteins were dialysed in a storage buffer (20 mM HEPES pH 7.5, 100 mM potassium acetate pH 7.5, 0.1 mM magnesium acetate, 10% glycerol, and 1 mM DTT) and flash frozen in the liquid nitrogen for further use.

### Fluorescence anisotropy analysis

The purified eIF2 complex or its mutant derivatives were serially diluted and incubated with a limiting concentration of 100 nM GDP-BODIPY (Invitrogen, #G22360) or GDPγS-BODIPY (Invitrogen, #22183) in a 1x anisotropy buffer (30 mM HEPES-KOH, pH 7.5, 110 mM Potassium acetate, 2.5 mM Magnesium acetate, 2 mM DTT and 0.6% glycerol) for 3 min at 26°C. Fluorescent polarization was measured on an iD5 multimode plate reader (Molecular Devices) using 495 nm excitation and 535 nm emission filters. The eIF2 complex or its mutant derivatives were carefully titrated to record the fluorescent polarization and converted into the anisotropy values. The fraction of GDP-BODIPY or GDPγS-BODIPY bound was calculated using the following formula.

$$f_{B}=\frac{r-r_{F}}{r_{B}-r_{F}}$$

Where *r* is the measured anisotropy, and *r*_F_ and *r*_B_ are the anisotropies of the free and saturated bound proteins, respectively. The *r*_F_ value is obtained by measuring the anisotropy of the free fluorophores as described earlier [49]. The values were fitted into a non-linear quadratic equation $y=V_{max}(x^{n}/k^{n}+x^{n})$ using Origin software. Where *V*_max_: maximum velocity, *K*: Michaelis’ constant, *n*: cooperative sites = 1

### 43-48S ribosome profile analysis

Yeast cells carrying WT or different derivatives of eIF2 mutants were grown in 250 ml SCD supplemented with uracil and histidine liquid culture till *A*_600_ ∼0.8. The liquid culture was snap-chilled on ice and mixed with ice-cold formaldehyde (1% final concentration) for 60 min. The formaldehyde crosslinking reaction was stopped by adding 0.1 M ice-cold glycine and incubating for 15 min on ice. Cells were harvested by centrifugation at 3000 × ***g*** for 5 min and washed twice with 1x lysis buffer (20 mM Tris-Cl pH 7.5, 50 mM KCl, 10 mM MgCl_2_, and 1 mM DTT). The cells were resuspended in 2 v/v of lysis buffer supplemented with a protease inhibitor cocktail, and cells were lysed mechanically with the glass beads. Cell debris was removed by centrifugation at 3000 × ***g*** for 5 min, and the supernatant was further clarified at 13,000 × ***g*** for 30 min at 4°C. A quantity of A_260_ ∼20 units from the WCE was layered on a 15%-40% sucrose density gradient and resolved by ultracentrifugation at 39,000 rpm (Beckman SW41) for 5 hrs at 4°C. Starting from the top to the bottom, 0.7 ml fractions were sequentially collected using the BioComp Inc fractionator. Fractions #7 and #8 containing 40-48S ribosomal subunits were taken for analysis. Each collected fraction was split into 0.25 and 0.45 ml. The 0.25 ml fraction was precipitated using two volumes of prechilled acetone on ice for 30 min. The pellet was obtained by spinning at 13,000 × ***g*** for 20 min at 4°C and washed with 70% prechilled ethanol. The pellet was dissolved in 25 µl of 2x SDS-Laemmli buffer, and part of it was resolved on 12% SDS-PAGE for Western blot analysis. For RNA extraction, the rest of the fractions (0.45 ml) were mixed with 900 μl of prechilled pure ethanol and 90 μl of 3M sodium acetate pH 5.0. The RNA was precipitated overnight at −20°C and pelleted down by centrifugation at 13000-× ***g*** for 30 min at 4°C. The pellet was resuspended in 300 μl of SDS-RNA lysis buffer (20 mM Tris-HCl pH 7.4, 100 mM NaCl, 1% SDS, and 2.5 mM EDTA) and RNA extraction was done twice with hot phenol (70°C) for 15 min in a thermomixer (1000 rpm) to reverse-crosslink the RNA. The aqueous phase containing RNA was precipitated by mixing with 500 μl ice-cold ethanol and 50 μl 3 M sodium acetate, pH 5.0. and incubating at −20°C for 2 h. The RNA was pelleted by centrifugation at 13,000 × ***g*** for 30 min at 4°C, and the pellet was washed with 70% ethanol. The air-dried pellet was reconstituted in 25 μl 2× RNA loading dye and part of it was resolved using 10% Urea-PAGE for Northern blot analysis as described above.

## Supplementary Material

Supplementary Figures S1-S4 and Supplementary Data

## Data Availability

All relevant data are included within the main article and its Supplementary File.

## Abbreviations

GAP: GTPase-activating protein
GDI: guanine nucleotide dissociation inhibitor
NTT: N-terminal tail
ORF: open reading frame
PIC: pre-initiation complex
SCD: synthetic complete dropout
SD: synthetic dextrose
ZBD: zinc-binding domain
