# Supplementary material for: eIF2β zinc-binding domain interacts with the eIF2γ subunit through the guanine nucleotide binding interface to promote Met-tRNAiMet binding
Source: Biosci Rep. 2024 Jul 5;44(7):BSR20240438. doi: 10.1042/BSR20240438 (PMC11230868; doi:10.1042/BSR20240438)
Supplement: Supplementary Figures S1-S4 and Supplementary Data [file BSR-2024-0438_supp.pdf]

Supplementary data;

Screening of the eIF2β^{S264Y} intragenic suppressor mutation

A single copy plasmid carrying the eIF2β^{S264Y} mutant gene was subjected to random mutagenesis using XL-1 Red *E. coli* mutagenic strain to prepare a library of mutant plasmids. We screened the library of mutant plasmids to identify mutations that suppressed the slow-growth phenotype associated with the eIF2β^{S264Y} mutation. Out of 1000 colonies screened, 7 colonies showed better growth phenotype than the original eIF2β^{S264Y} mutation. Sequencing of the plasmids isolated from these 7 colonies revealed two revertant mutations, one mutation converted the original eIF2β^{S264Y} residue to alanine, and four plasmids showed eIF2β-Thr238 to Ala change.

Figure legend (supplementary figures)

Figure S1. Co-immunoprecipitation of 3HA-tagged eIF2β subunit.

Yeast strain YP896 (*his4Δ*, *sui3Δ*) carrying single copy YCplac111_HA_SUI3 (A202) or YCplac111_HA_SUI3-T238A (A1394) plasmids were grown overnight. The whole cell extract (WCE) was prepared by mechanical cell breaking using glass beads. The N-terminal end of the eIF2β protein was fused with a 3xHA tag, and using anti-HA antibody agarose beads, the eIF2β protein was coimmunoprecipitated from the WCE and subjected to the Western blotting using an anti-HA antibody (eIF2β), anti-eIF2γ, anti-eIF2α, anti-eIF2Bε , and RPS20 antibodies. Half of the Co-IP beads were treated with Trizol, and the RNA was precipitated with ethanol and subjected to the Northern blot analysis using a probe specific to the initiator Met-tRNA$_i$. Full membrane images of Western and Northern blot showing red highlighted region (bottom).

Figure S2. Analysis of TC on the 43-48S ribosome. Yeast strain YP912 (*sui3Δ, gcd11Δ*) carrying derivatives of eIF2β mutant were grown overnight A_{600} ~0.8. The WCE (A_{260} ~20 U) was layered on a 15%-40% sucrose gradient and resolved by ultracentrifugation at 39,000 rpm (Beckman SW41 rotor) for 5 hrs at 4°C. A 0.7 ml of 15 factions was collected by continuously monitoring A_{260} nm (Polysome profile: top panel: A). Part of these factions was resolved on the 12% SDS-PAGE (middle panel: B) and analyzed by the Western blotting using indicated antibodies or by Northern blot to identify Met-tRNA$_i$ (panel C), Fraction #6-9 (dotted region) were re-ran on separate gels and analyzed by Western or Northern blot to identify 40S subunit fractions (panel: D), Fractions #7 and #8 were identified to contain the majority of 40S-48S complexes. Using these parameters, the yeast strain YP912 carrying derivatives of eIF2β mutant were subjected to 1% HCHO cross-linking as described in Materials and Methods and fractionated as described above. The fractions #1-12 were analysed by Western blot using indicated antibodies or Northern blot to identify Met-tRNA$_i$. The fractions #7 and #8 containing 40S-48S complexes is marked with the dotted lines that shows the corresponding ribosome profile at

the bottom. In; is input. Full membrane images (Western and Northern blot showing red highlighted region) of TC on the 43-48S ribosome (bottom or right side).

Figure S3. Analysis of the purified WT and mutant eIF2 complex. The eIF2 complexes were purified from strains overexpressing eIF2αβγ subunits as described in Materials and Methods. Different amounts (0.5 and 2 μg) of the eIF2 complexes were resolved by 12% sodium dodecyl sulfate-polyacrylamide gel electrophoresis and visualized by Western blot (top) or Coomassie blue stain (bottom). The positions of the eIF2γ, eIF2α , and eIF2β subunits are shown on the right. Full membrane images of Western blot showing red highlighted region (bottom).

Figure S4. The eIF2β^{T238A} mutation suppresses the Sui^{-} and Gcd^{-} phenotype of the eIF2β HTH mutants.

A) Growth analysis. Yeast strain YP896 (*his4Δ*, *sui3Δ*) carrying single copy eIF2β^{WT} (A1451), eIF2β^{F217A,Q221A} (A1490), eIF2β$^{F217A,Q221A/T238A}$ (A1492) or eIF2β^{T238A} (A1260) plasmids were grown overnight, serially diluted, and spotted on SD plus uracil, tryptophan, and histidine plate and incubated at 30°C for 2–3 days. B) Analysis of *HIS4-lacZ* expression. Yeast strains from (A) were transformed with either A1072 (GAPDH$_{prom}$_His4^{AUG}_lacZ) or A1073 (GAPDH$_{prom}$_His4^{UUG}_lacZ) plasmid constructs and grown on the SCD plus tryptophan media and harvested at OD_{600} ~ 0.8. The harvested cells were subjected to β-galactosidase assay (nmol of O-nitrophenyl-β-D-galactopyranoside cleaved per min per mg), and the resultant values were plotted as UUG/AUG ratio. C) Analysis of *GCN4-lacZ* expression. Yeast strains from (A) were transformed with *GCN4-lacZ* construct (p180). The measurement of β-galactosidase activity was done as described above. Statistical differences were determined by one-way ANOVA analysis. The error bar shows the standard deviation.

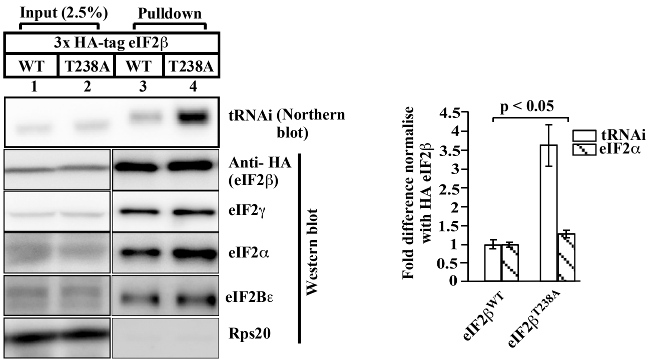

Figure S1

Co-Immunoprecipitation of 3HA-tagged eIF2β subunit.

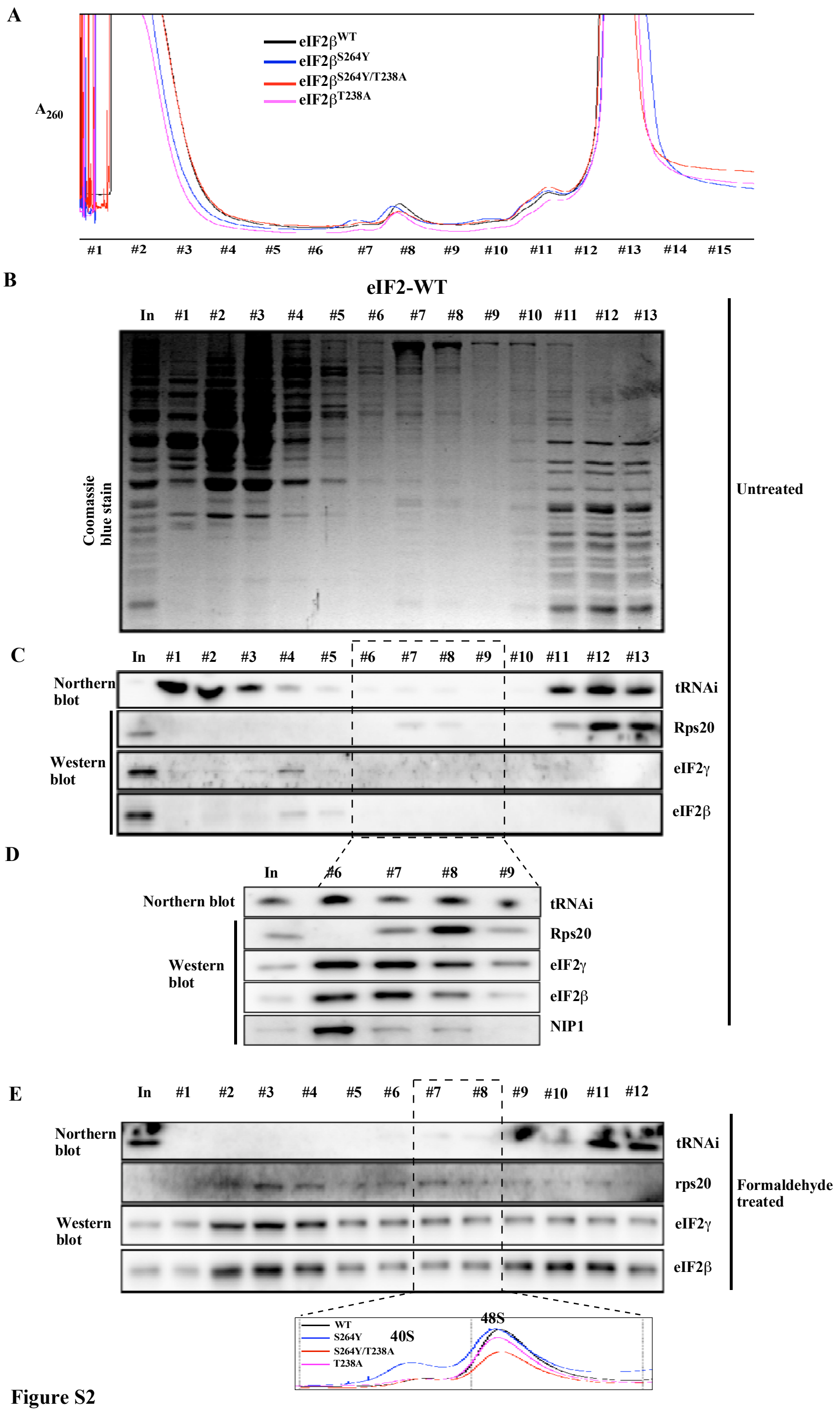

Figure S2

Analysis of TC on the 43-48S ribosome.

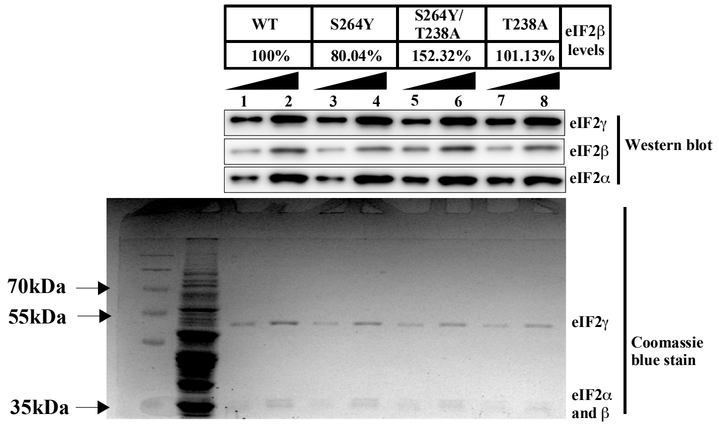

Figure S3
Analysis of the purified WT and mutant eIF2 complex.

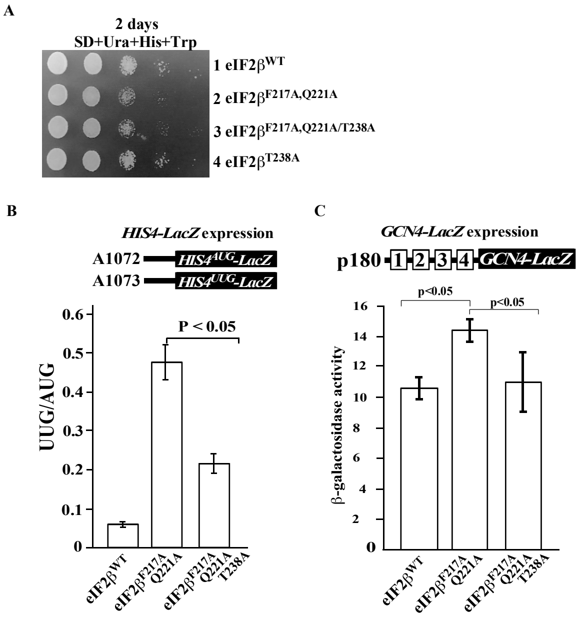

Figure S4

The eIF2β^{T238A} mutation suppresses Sui^{-} and Gcd^{-} phenotype of the eIF2β HTH mutants
